# Viridistratins A−C, Antimicrobial and Cytotoxic Benzo[*j*]fluoranthenes from Stromata of *Annulohypoxylon viridistratum* (Hypoxylaceae, Ascomycota)

**DOI:** 10.3390/biom10050805

**Published:** 2020-05-23

**Authors:** Kevin Becker, Anna-Charleen Wessel, J. Jennifer Luangsa-ard, Marc Stadler

**Affiliations:** 1 Department of Microbial Drugs, Helmholtz Centre for Infection Research GmbH (HZI), Inhoffenstraße 7, 38124 Braunschweig, Germany; kevin.becker@helmholtz-hzi.de (K.B.); anna-charleen.wessel@helmholtz-hzi.de (A.-C.W.); 2 German Centre for Infection Research Association (DZIF), Partner site Hannover-Braunschweig, Inhoffenstraße 7, 38124 Braunschweig, Germany; 3 National Center for Genetic Engineering and Biotechnology (BIOTEC), 113 Thailand Science Park, Phahonyothin Rd., Khlong Nueng, Khlong Luang, Pathum Thani 12120, Thailand; jajen@biotec.or.th

**Keywords:** Ascomycota, benzo[*j*]fluoranthenes, chemotaxonomy, chromatography, secondary metabolites, structure elucidation, Xylariales

## Abstract

During the course of our search for novel biologically active metabolites from tropical fungi, we are using chemotaxonomic and taxonomic methodology for the preselection of interesting materials. Recently, three previously undescribed benzo[*j*]fluoranthenes (**1**−**3**) together with the known derivatives truncatones A and C (**4**, **5**) were isolated from the stromata of the recently described species *Annulohypoxylon viridistratum* collected in Thailand. Their chemical structures were elucidated by means of spectral methods, including nuclear magnetic resonance (NMR) spectroscopy and high-resolution mass spectrometry (HR-MS). The new compounds, for which we propose the trivial names viridistratins A−C, exhibited weak-to-moderate antimicrobial and cytotoxic activities in cell-based assays.

## 1. Introduction

The genus *Annulohypoxylon* belongs to the fungal family Hypoxylaceae (order Xylariales), which was recently resurrected [1] to accommodate *Hypoxylon* and allied genera and is known for a remarkably diverse secondary metabolism [2]. It was segregated from *Hypoxylon* by Hsieh et al. in 2005 [3], and its type species, *Annulohypoxylon truncatum,* was previously known as *Hypoxylon truncatum*. In the past 15 years, these fungi have been the subject of intensive studies of their secondary metabolites, revealing an enormous diversity of bioactive compounds [4]. In particular, their stromatal pigments are of chemotaxonomic value [2,5]. Several studies of old types of material of *Hypoxylon* and allied genera using high-performance liquid chromatography coupled to diode array detection and mass spectrometry (HPLC-DAD/MS) have revealed that those pigments can remain stable for centuries [5,6,7]. A recent study on *Hypoxylon fragiforme* showed that these pigments can even endure many centuries and remain intact in fossil samples [7]. Two classes of secondary metabolites are predominant in the stromata of the Hypoxylaceae, i.e., azaphilones and naphthalene derivatives [8]. Recent major phylogenetic studies [1,9], which were now even confirmed by phylogenomics [10] have led to the segregation of the new genus *Jackrogersella* (with *J. multiformis*, *J. cohaerens* and *J. minutella* being examples of species that contain azaphilones as predominant stromatal pigments), which was segregated from *Annulohypoxylon* based on a combination of morphological and chemotaxonomic traits with the results of a multilocus molecular phylogeny. These azaphilones of *Jackrogersella* are of the cohaerin type, which also includes the multiformins and minutellins that are derived from the same unique polyketide scaffold [7,10,11]. Examples of common naphthalene derivatives in *Annulohypoxylon* spp. are the rather ubiquitous 1,1’-binaphthalene-4,4’,5,5’-tetrol (BNT) [2], as well as oxidized derivatives thereof, such as truncatones [2,12], hypoxylonols [2,13,14] and daldinones [2,11], with the three latter ones belonging to the subclass of benzo[*j*]fluoranthenes. The latter compound class has been exclusively found in *Annulohypoxylon* species and was so far not detected in *Jackrogersella* and *Hypoxylon* [2,4], even though binaphthalenes also occur in other, more distantly related Hypoxylaceae such as *Daldinia* [5].

Among the species that were only recently described in 2017 [2], *Annulohypoxylon viridistratum* was reported to have a unique secondary metabolite profile. Several yet unknown compounds were detected in the holotype specimen by database-aided HPLC-DAD/MS detection (see [2] and Figure S3). While the valuable holotype specimen was not suitable for preparative work, also because the stromatal material was rather scarce, we were recently able to collect another specimen in the field, which turned out to represent the same species and provided us with sufficient amounts of material to envisage the isolation of these unknown compounds. The current paper is dedicated to the description of their isolation, structure elucidation and biological activities.

## 2. Materials and Methods

### 2.1. General

Electrospray mass (ESI-MS) spectra were recorded with an UltiMate^®^ 3000 Series uHPLC (Thermo Fisher Scientific, Waltman, MA, USA) utilizing a C18 Acquity^®^ UPLC BEH column (2.1 × 50 mm, 1.7 µm; Waters, Milford, USA) connected to an amaZon speed^®^ ESI-Iontrap-MS (Bruker, Billerica, MA, USA). HPLC parameters were set as follows: solvent A: H_2_O+0.1 % (*v*/*v*) formic acid, solvent B: acetonitrile (ACN)+0.1 % (*v*/*v*) formic acid, gradient 5 % B for 0.5 min, increasing to 100 % B in 19.5 min, keeping 100% B for further 5 min, flowrate 0.6 mL/min, and DAD detection 190–600 nm.

High-resolution electrospray mass (HR-ESI-MS) spectra were obtained with an Agilent 1200 Infinity Series HPLC (Agilent Technologies, Santa Clara, CA, USA) connected to a maXis^®^ electrospray time-of-flight mass spectrometer (ESI-TOF-MS; Bruker; HPLC conditions same as for ESI-MS spectra).

Nuclear magnetic resonance (NMR) spectra were recorded with an Avance III 500 spectrometer (Bruker, ^1^H NMR: 500 MHz, and ^13^C NMR: 125 MHz). UV/Vis spectra were taken with a UV/Vis spectrophotometer UV-2450 (Shimadzu, Kyoto, Japan), and electronic circular dichroism (ECD) spectra (Figure S2) were recorded on a J-815 spectropolarimeter (JASCO, Pfungstadt, Germany).

### 2.2. Fungal Material

Stromata of *Annulohypoxylon viridistratum* were collected by L. Wendt from unidentified dead wood in a tropical rainforest in Thailand, Nan Bo Kluea, Khun Nan National Park (19.18301 N, 101.17801 E) in August of 2015 during the rainy season. A voucher specimen, which showed the characteristics of the species, was identified by E. Kuhnert and L. Wendt by a comparison of morphological characteristics, as well as molecular phylogenetic studies and HPLC profiling [2]. It is deposited in the herbarium of the National Center for Genetic Engineering and Biotechnology (BIOTEC), 113 Thailand Science Park, Phahonyothin Rd., Khlong Nueng, Khlong Luang, Pathum Thani 12120, Thailand (acc. No. BBH40533). This specimen is only the second record of this recently described species.

### 2.3. Extraction and Isolation

The dried stromata (1.45 g) were extracted a total of three times: (1) The stromata were carefully scraped off the dead wood and extracted with 100 mL of acetone in an ultrasonic bath (40°C, 60 min). The extract was centrifuged (4000 rpm, 5 min), and the supernatant transferred to a round-bottom flask. (2) The precipitate was extracted again with 100 mL of acetone, centrifuged and the supernatant transferred as described before. (3) Finally, the precipitate was crushed utilizing a mortar and pestle before being treated as in step (2) again. The supernatants of all three extractions were combined and dried in vacuo at 40°C, which yielded the crude extract (227.6 mg). The stromatal remnants were discarded.

The crude extract was prefractionated with a Strata^®^ C18-E cartridge (10 g/60 mL, 55 µm, 70 Å; P/N: 8B-S001-MFF, Phenomenex, Torrance, CA, USA). At first, the sample was dispersed in water and added to the cartridge. Applying a vacuum of ca. 600 mbar removed the water while leaving the sample on the cartridge. Then, a step gradient of the solvent waters (solvent A), acetonitrile (ACN; solvent B) and acetone (solvent C) was applied with the following steps: (I) 80:20:0 (A:B:C, % *v*/*v*/*v*), (II) 50:50:0, (III) 0:100:0, (IV) 0:50:50 and (V) 0:0:100. For each step, 40 mL of solvent was used, and elution was aided by applying a vacuum (600 mbar), which yielded fractions I−V. 

Fraction III (83.3 mg) was further processed using a PLC 2250 system (Gilson, Middleton, WI, USA). The sample was dissolved in 3 mL acetone:H_2_O:ACN 4:1:1 (*v*/*v*/*v*). A Nucleodur^®^ C18ec column (125×40 mm, 7 µm, 100 Å; Machery-Nagel, Düren, Germany) was utilized using H_2_O+0.1 % (v/v) formic acid (solvent A) and ACN+0.1 % (*v*/*v*) formic acid (solvent B) as eluents. Using a flow rate of 50 mL/min, a gradient was applied from 35 % to 80 % B within 50 min, followed by an increase of 80 % to 100 % B in 10 min, followed by another 10 min of 100 % B. Fractions were taken every 20 mL.

This yielded the pure compounds **1**−**5**: viridistratin A (**1**, fractions #36−41, 3.2 mg), truncatone C (**5**, #42−49, 9.6 mg), truncatone A (**4**, #59−61, 3.4 mg), viridistratin B (**2**, #70−80, 17.5 mg) and viridistratin C (**3**, #99−106, 8.0 mg).

### 2.4. Antimicrobial Activity Assay

Compounds **1**, **2**, **4** and **5** were dissolved in MeOH (1 mg/mL) for the antimicrobial activity assay, while compound **3** was dissolved in MeOH: DMSO 9+1 (1 mg/mL). The solvents were also used as negative controls.

Minimum inhibitory concentrations (MIC) were determined in a serial dilution assay, as described previously [15]; a detailed protocol can be found in the Supporting Information. The compounds (1 mg/mL) were diluted to a range of 66.7 to 0.52 µg/mL and incubated with the test organisms overnight. Inhibition of growth was visually evaluated the next day: the MIC is defined as the lowest concentration of the test compound where no growth of the test organism was observed. Various test organisms of fungal and bacterial origin were tested to cover a broad range of microorganisms. This selection is also being used as a standard test panel in our attempts to discover new anti-infectives, as it represents a broad spectrum of pathogens of clinical interest, as well as sensitive indicator strains (bacteria: *Bacillus subtilis*, *Staphylococcus aureus*, *Micrococcus luteus*, *Chromobacterium violaceum*, *Escherichia coli* and *Pseudomonas aeruginosa*; mycobacteria: *Mycolicibacterium smegmati*s and fungi: *Candida albicans*, *Schizosaccharomyces pombe*, *Mucor hiemali*s, *Pichia anomala* and *Rhodotorula glutinis*).

### 2.5. Cytotoxicity Assay

Compounds **1**−**5** were dissolved as described in the previous section. The cytotoxicity assay was initially performed against the cell lines L929 (mouse fibroblasts), as well as KB 3.1 (human papillomavirus-related endocervical adenocarcinoma), as described previously [16]. A detailed protocol, as well as sources of the cell lines, is given in the Supporting Information.

After incubating the cell lines with a serial dilution of the test compounds (final range: 37 to 0.6 × 10^−3^ µg/mL) for five days, the cells were dyed using 3-(4,5-dimethyl-2-thiazolyl)-2,5-diphenyl-2*H*-tetrazolium bromide (MTT), which is only converted to its purple formazan derivative by living cells. Then, the intensity of the purple derivative in relation to cells without additive (set to 100% viability) for each concentration of a test compound was quantified. For this, the absorption at 595 nm was measured using a microplate reader to calculate the percentage of cell viability. From this, the half-maximum inhibitory concentration (IC_50_, in µM) was calculated. 

If an inhibition of cell viability with an IC_50_ < 50 µM was observed, further cell lines were subjected to the test compounds: PC-3 (human prostate adenocarcinoma), SK-OV-3 (human ovary adenocarcinoma), MCF-7 (human breast adenocarcinoma), A431 (human squamous carcinoma) and A549 (human lung carcinoma).

### 2.6. Spectral Data

#### 2.6.1. Viridistratin A (**1**)

Yellow powder. NMR (acetone-*d_6_*, ^1^H NMR: 500 MHz, ^13^C NMR: 125 MHz): see Table 1; UV/Vis (c = 0.01 mg/mL, ACN): λ_max_ (*ε*) = 251 (4.45), 342 (4.14), 350 (4.12), 406 (3.94) and 428 (3.91) nm; ESI-MS: *m/z* 301.06 (M+H)^+^ and 298.94 (M−H)^−^; HR-ESI-MS: *m/z* 301.0855 (M+H)^+^ (calculated for C_20_H_13_O_3_, 301.0859); t*_R_*= 9.3 min.

#### 2.6.2. Viridistratin B (**2**)

Yellow powder. NMR (acetone-*d_6_*, ^1^H NMR: 500 MHz, ^13^C NMR: 125 MHz): see Table 1; UV/Vis (c = 0.01 mg/mL, ACN): λ_max_ (*ε*) = 248 (4.64), 327 (4.23), 341 (4.38), 384 (4.01) and 403 (4.07) nm; ESI-MS: *m/z* 315.10 (M+H)^+^ and 312.96 (M−H)^−^; HR-ESI-MS: *m/z* 315.1010 (M+H)^+^ (calculated for C_21_H_15_O_3_, 315.1016); t*_R_*= 12.2 min.

#### 2.6.3. Viridistratin C (**3**)

Yellow powder. NMR (DMSO-*d_6_*, ^1^H NMR: 500 MHz and ^13^C NMR: 125 MHz): see Table 1; UV/Vis (c = 0.01 mg/mL, CHCl_3_): λ_max_ (*ε*) = 326 (4.18), 341 (4.46), 384 (3.91) and 403 (3.97) nm; ESI-MS: *m/z* 329.12 (M+H)^+^ and 327.01 (M−H)^−^; HR ESI-MS: *m/z* 329.1168 (M+H)^+^ (calculated for C_22_H_17_O_3_, 329.1172); t*_R_*= 13.9 min.

## 3. Results

### 3.1. Structure Elucidation of Viridistratins A−C (**1**−**3**)

**Figure 1:**
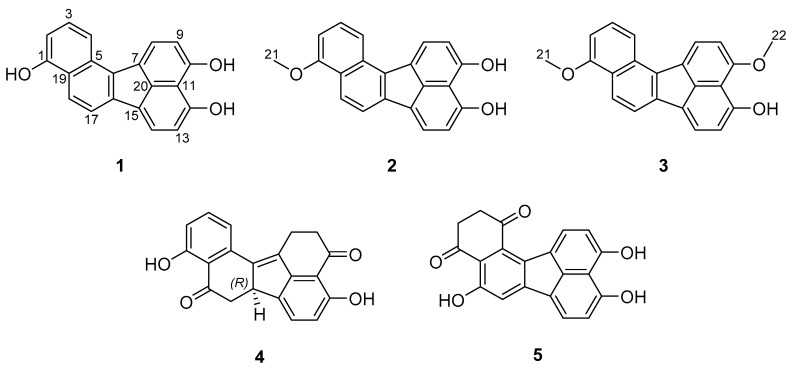
Structures of secondary metabolites isolated from stromata of *Annulohypoxylon viridistratum*. **1**−**3**: viridistratins A−C, **4**: truncatone A and **5**: truncatone C.

In total, five compounds were isolated from the stromatal extract of *A. viridistratum* in substantial amounts. Three of them represent novel secondary metabolites for which we propose the trivial names viridistratins A−C (**1**−**3**) (see Figure 1), while the remaining two compounds were identical to the previously reported truncatones A (**4**) and C (**5**) [12].

Viridistratin A (**1**) was isolated as a yellow powder and shown to possess a molecular formula of C_20_H_12_O_3_ by HR-ESI-MS, which corresponds to 15 double-bond equivalents. In combination with the UV/Vis maxima (see Figure S1), a large, highly conjugated aromatic system was indicated. In the ^1^H NMR and ^1^H/^13^C heteronuclear single quantum coherence spectroscopy (^1^H/^13^C-HSQC) spectra, nine aromatic methines were observed. Additionally, the ^13^C NMR and ^1^H/^13^C heteronuclear multiple-bond correlation spectroscopy (^1^H/^13^C HMBC) spectra showed the presence of eleven sp^2^-hybridized carbons. Three of those eleven carbons had chemical shifts indicating a linkage to a hydroxy group. Analysis of the ^1^H/^1^H correlation spectroscopy (^1^H/^1^H COSY) spectra revealed the presence of four separate spin systems: (1) 1-H, 2-H and 3-H; (2) 8-H and 9-H and (3) 13-H and 14-H, as well as (4) 17-H and 18-H, which was supported by their splitting patterns in the ^1^H NMR spectrum. Analysis of the ^1^H/^13^C HMBC correlations revealed two naphthalene moieties linked to each other by two C-C bonds to form a five-membered ring in between (i.e., a benzo[*j*]fluoranthene backbone). Three hydroxy groups were connected to the core structure by analysis of the proton and carbon shifts of the neighboring atoms.

**Figure 2:**
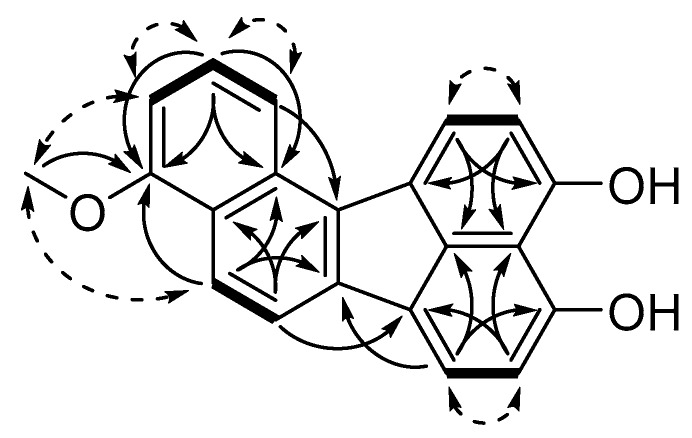
Key nuclear magnetic resonance (NMR) correlations of viridistratin B (**2**). Bold bonds: ^1^H/^1^H correlation spectroscopy (COSY) correlations, plain arrows: ^1^H/^13^C (heteronuclear multiple bond correlation (HMBC) correlations and dashed arrows: rotating frame nuclear Overhauser effect spectroscopy (ROESY) correlations.

Viridistratin B (**2**) was isolated as a yellow powder and showed a molecular formula of C_21_H_14_O_3_, indicating a formal addition of CH_2_ compared to **1**. An additional singlet in the ^1^H and ^1^H/^13^C HSQC NMR spectra (21-H_3_, δ_H_ = 4.04), as well as a ^1^H/^13^C HMBC correlation of 21-H_3_ to C-1, revealed **2** to be the 1-methoxy derivative of **1**. Key ^1^H/^1^H COSY, ^1^H/^13^C HMBC and rotating frame nuclear Overhauser effect spectroscopy (ROESY) correlations of **2** are depicted in Figure 2 as an example for the new compounds isolated in this work.

Viridistratin C (**3**) was isolated as a yellow powder and shown to have a molecular formula of C_22_H_16_O_3_, indicating yet another addition of a methylene in comparison to **2**. The ^1^H/^13^C HMBC correlations of the singlet 22-H_3_ (δ_H_ = 4.09) to C-10 showed their linkage via the hydroxy group OH-10. Eventually, **3** is the 1,10-dimethoxy derivative of **1**.

Structures of truncatones A and C (**4**, **5**) were elucidated by NMR spectroscopy and verified with data provided in the original publication by Sudarman et al. [12]. The stereochemistry of truncatone A was confirmed by the comparison of taken electronic circular dichroism (ECD) spectra (see Figure S2) with data published therein.

### 3.2. Antibacterial, Antifungal and Cytotoxic Activities of Compounds **1**−**5**

The minimum inhibitory concentrations (MIC) of **1**−**5** were assessed as described in the Methods Section, and the results are summarized in Table 2. For simplified evaluation, measured MIC values were assigned to three descriptors whose thresholds were derived from the measured MIC of the references: strong (MIC_compound_ < MIC_Reference_), moderate (MIC_compound_ ≈ MIC_Reference_) and weak activity (MIC_compound_ > MIC_Reference_).

Viridistratin A (**1**) exhibited weak antibacterial activities against Gram-positive bacteria, with the strongest activity against *Micrococcus luteus* (16.7 µg/mL). In addition, growth of the Gram-negative *Chromobacterium violaceum* was inhibited at 66.7 µg/mL. Furthermore, moderate antifungal activity against all tested fungi except *Candida albicans* was observed. Viridistratin B (**2**) showed a similar antimicrobial activity spectrum, additionally being able to inhibit the growth of *Mycolicibacterium smegmatis* (33.3 µg/mL). In comparison to **1**, compound **2** was generally more active with the lowest MIC of 8.3 µg/mL against *Micrococcus luteus,* as well as a strong inhibition of *Mucor hiemalis* (4.2 µg/mL). Viridistratin C (**3**) and truncatone A (**4**) only exhibited weak antibacterial activity against *Micrococcus luteus* (33.3 and 16.7 µg/mL, respectively), while, for truncatone C (**5**), weak activities against *Bacillus subtilis* and *Micrococcus luteus* with MIC of 66.7 and 16.7 µg/mL were observed. Truncatone C additionally exhibited weak antifungal activity against *Mucor hiemalis* and moderate activity against *Rhodotorula glutinis* (66.7 and 16.7 µg/mL)

Concerning the cytotoxicity of **1**−**5**, an effect of all compounds against the chosen cell lines was measured and is summarized in Table 3. Against the mouse fibroblast cell line L929, truncatone A (**4**) and viridistratin A (**1**) showed half-maximum inhibitory concentration (IC_50_) values of 10.4 µM (3.3 µg/mL) and 12.7 µM (8.5 µg/mL), respectively. Against human cell lines, viridistratin B (**2**) induced a decrease of cell viability at IC_50_ values of 1.1 µM (0.34 µg/mL) and 1.4 µM (0.45 µg/mL) against A431 and A549. Compounds **1**, **4** and **5** exhibited a reduced cytotoxicity (i.e., a higher IC_50_) as compared to **2** and **3**. Viridistratin C (**3**) showed a low decrease in cell viability (IC_50_ > 50 µM) against L929 and KB 3.1 and was excluded from tests against further cell lines.

To conclude, it can be summarized that the strongest antimicrobial activities (lowest MIC) among the tested compounds, as well as the strongest cytotoxicities (lowest IC_50_), were both exhibited by viridistratin B (**2**). In general, viridistratin C (**3**) showed the highest MIC values, as well as the highest IC_50_. The other compounds tested (**1**, **4** and **5**) lied in between.

## 4. Discussion

The three secondary metabolites viridistratins A−C (**1**−**3**) described in this study constitute a new subclass of benzo[*j*]fluoranthenes from stromata of *Annulohypoxylon,* whose backbone only consists of aromatic carbons. Due to their aromaticity, no stereochemistry is present in those metabolites.

Peaks corresponding to the viridistratins were already detected by Kuhnert et al. from the holotype specimen and designated UCV1−3 [2]. Occurrence of those compounds has not been observed in any other *Annulohypoxylon* species yet, even though over two-hundred specimens, including almost all holotypes, have already been examined [2]. Thus, **1**−**3** can serve as chemotaxonomic markers for the distinction of *A. viridistratum* from other *Annulohypoxylon* spp. Along with the viridistratins, the truncatones A (**4**) and C (**5**) were isolated, which are common metabolites in *Annulohypoxylon* spp. and found in many representatives [2]. Interestingly, truncatone A (**4**) was not detected in crude extracts of the stromata and, thus, may be a conversion product from the isolation process (see Figure S3). In general, it should be mentioned that all benzo[*j*]fluoranthenes hitherto isolated from Xylariales were exclusively obtained from members of the genus *Annulohypoxylon* in the current sense. Previous reports on the isolation of these compounds from “*Hypoxylon*” *truncatum* (which is now the type species of *Annulohypoxylon* [3]) actually may go back to this species or its morphologically similar relatives. The genus *Hypoxylon* sensu Wendt et al. [1], as well as the species of the recently erected genus *Jackrogersella,* are characterized by the lack of this compound class as stromatal metabolites, and this chemotaxonomic evidence has strongly supported the recent rearrangement of the genera of the Hypoxylaceae.

Sudarman et al. [12] postulated the benzo[*j*]fluoranthenes to be derived from the 1,8-DHN (dihydroxy naphthalene) pathway [17] of the melanin biosynthesis, with BNT as the precursor for further oxidations. Following this hypothesis, the complete absence of BNT in stromatal extracts of *A. viridistratum* means that BNT was fully converted to its biosynthetical successors. The origin of these aromatic compounds from the 1,8-DHN pathway has been established in other Ascomycota, and the corresponding polyketide biosynthetic gene clusters are thought to be derived from the 1,8-DHN melanin pathway [18]. However, so far, the biosynthetic genes for these secondary metabolites have not been identified for any species of *Annulohypoxylon*. This can only now be made possible, because high-quality genomes of several Hypoxylaceae, including the type species of *Annulohypoxylon*, have recently become available for the first time [19].

Viridistratins **1**−**3** exhibited antimicrobial activity against bacteria and fungi but with moderate-to-weak effects. Especially *Micrococcus luteus* was sensitive to the tested benzo[*j*]fluoranthenes. Among the tested compounds, vidiristratin B (**2**) showed the highest activities. Notably, growth of *Mucor hiemalis* was inhibited by **2** at comparably low concentrations of 4.2 µg/mL. For related benzo[*j*]fluoranthenes like truncatones [10], hypoxyonols [13,14] or daldinols [20,21], no antimicrobial assessments have been reported in the literature. Furthermore, viridistratin B (**2**) showed the lowest half-maximum inhibitory concentrations (IC_50_) against all human cell lines, followed by **1**, **4** and **5**, while viridistratin C (**3**) showed only a weak decrease of mammalian cell viability. Other related benzo[*j*]fluoranthenes like truncatones A, C and D were previously shown to be cytotoxic against the KB 3.1 and L929 cell lines, even though truncatones were reported to exhibit lower IC_50_ values [12]. Hypoxylonol C was shown to have cell-protective effects against stereptozotocin-induced damage in INS-1 cells [22], while hypoxylonol F was observed to improve insulin secretion [23]. These two reports of rather beneficial bioactivities, although apparently contradicting the cytotoxic activities of hypoxylonols A−F published before [13,14], can be explained by the usage of different doses, as well as target systems. Daldinols C and D were also shown to be cytotoxic against human colon adenocarcinoma cells [21]. Hence, it can be assumed that cytotoxic activities of the aforementioned compounds, as well as the new viridistratins, arise from the benzo[*j*]fluoranthene backbone, which is well-known for its cytotoxic and cancerogenic properties [24]. These properties arise from the hydrophobicity of the compounds, which reduce (essential) structural interactions between cellular macromolecules such as lipid layers or enzymes by inducing water stress [25,26]. These unspecific targets of such hydrophobic molecules consequently explain the measured bioactivity of substances such as viridistratins A−C (**1**−**3**) against diverse mammalian, bacterial, and fungal cells.

## 5. Conclusions

The novel compounds viridistratins A−C (**1**−**3**) described herein constitute a novel subclass of hydroxylated benzo[*j*]fluoranthenes with varying methoxylation levels. So far, they have exclusively been detected in *A. viridistratum* and may thus serve as chemotaxonomic markers for the species. Chemotaxonomic markers are the basis for HPLC fingerprinting [2], which can help to aid in the discrimination of morphologically similar species.

Several other benzo[*j*]fluoranthenes have been described from *Annulohypoxylon* spp. already [12,13,14,21], all of which possess cytotoxic activity against various mammalian cell lines. Viridistratins A−C additionally exhibited weak antibacterial and antifungal activities, with the lowest minimum inhibitory concentrations (MIC) against *Micrococcus luteus* and *Mucor hiemalis*. The broad range of activity suggests a nonselective mode of action against the tested microorganisms and mammalian cell lines.

Therefore, and because of the scarce availability and the instability observed in some derivatives, the viridistratins and related metabolites do not appear attractive for further developments as drugs, but they may have a protective function for the producer organisms in nature.

## Figures and Tables

**Table 1:** 1D nuclear magnetic resonance (NMR) data of **1**−**3** (**1**, **2**: acetone-*d_6_* and **3**: DMSO-*d_6_*; ^1^H NMR: 500 MHz and ^13^C NMR: 125 MHz).

pos ^1^	1			2			3	
	δ_C_, mult ^2^	δ_H_, mult ^2^		δ_C_, mult	δ_H_, mult		δ_C_, mult	δ_H_, mult
1	155.1, C			156.8, C			156.2, C	
2	108.2, CH	6.90, d (7.53)		103.6, CH	6.92, d (7.63)		104.0, CH	6.97, m
3	128.1, CH	7.42, t (2 × 7.96)		127.5, CH	7.52, t (2 × 8.09)		127.9, CH	7.56, t (2 × 7.96)
4	116.4, CH	8.22, d (8.39)		116.8, CH	8.29, d (8.39)		116.6, CH	8.30, d (8.39)
5	132.5, C			131.5, C			130.9, C	
6	133.5, C			133.0, C			131.6, C	
7	129.9, C			129.3, C			129.2, C	
8	127.4, CH	8.45, m		126.9, CH	8.45, d (7.78)		126.8, CH	8.56, d (8.17)
9	111.2, CH	7.06, d (7.53)		110.8, CH	7.06, d (7.78)		106.7, CH	7.13, d (7.96)
10	156.2, C			155.7, C			158.1, C	
11	112.6, C			112.1, C			112.7, C	
12	156.8, C			156.3, C			156.8, C	
13	110.9, CH	7.01, d (7.53)		110.5, CH	7.01, d (7.63)		112.0, CH	7.00, m
14	124.3, CH	8.06, br s		123.8, CH	8.05, m		124.6, CH	8.09, m
15	129.1, C			128.5, C			126.7, C	
16	137.9, C			137.5, C			137.3, C	
17	119.0, CH	8.06, m		118.9, CH	8.06, m		119.2, CH	8.09, m
18	121.8, CH	8.26, d (8.60)		120.8, CH	8.22, m		120.7, CH	8.17, d (8.60)
19	125.4, C			125.4, C			124.5, C	
20	135.2, C			134.7, C			134.1, C	
21				55.5, CH_3_	4.04, s		56.0, CH_3_	4.02, s
22							56.8, CH_3_	4.09, s

^1^ pos: atom position (see Figure 1); ^2^ δ_C_/δ_H_: chemical shift [ppm]; mult: multiplicity, (br) s: (broad) singlet, d: doublet, t: triplet, and m: multiplet.

**Table 2:** Minimum inhibitory concentrations (MIC) of **1**−**5** against bacterial and fungal test organisms.

Test Organism	Minimum Inhibitory Concentration (MIC) (µg/mL)					
	1	2	3	4	5	Reference
*Bacillus subtilis*	33.3	16.7	>66.7	>66.7	66.7	8.3 ^1^
*Staphylococcus aureus*	66.7	16.7	>66.7	>66.7	>66.7	0.4 ^2^
*Micrococcus luteus*	16.7	8.3	66.7	33.3	16.7	0.8 ^2^
*Chromobacterium violaceum*	66.7	66.7	>66.7	>66.7	>66.7	0.1 ^2^
*Escherichia coli*	>66.7	>66.7	>66.7	>66.7	>66.7	1.7 ^2^
*Pseudomonas aeruginosa*	>66.7	>66.7	>66.7	>66.7	>66.7	0.4 ^3^
*Mycolicibacterium smegmatis*	>66.7	33.3	>66.7	>66.7	>66.7	3.3 ^4^
*Candida albicans*	>66.7	>66.7	>66.7	>66.7	>66.7	66.7 ^5^
*Schizosaccharomyces pombe*	66.7	33.3	>66.7	>66.7	>66.7	33.3 ^5^
*Mucor hiemalis*	66.7	4.2	>66.7	>66.7	66.7	66.7 ^5^
*Pichia anomala*	66.7	33.3	>66.7	>66.7	>66.7	66.7 ^5^
*Rhodotorula glutinis*	33.3	33.3	>66.7	>66.7	66.7	16.7 ^5^

^1^ oxytetracycline 1 mg/mL, ^2^ oxytetracycline 0.1 mg/mL, ^3^ gentamicin 0.1 mg/mL, ^4^ kanamycin 0.1 mg/mL and ^5^ nystatin 1 mg/mL.

**Table 3:** Cytotoxicity of **1**−**5** against mammalian cell lines as half-maximum inhibitory concentrations (IC_50_). n.d.: not determined.

Cell Line		Cytotoxicity (IC_50_) (µM)					
		1	2	3	4	5	Reference ^1^
L929	mouse fibroblasts	12.7	17.2	61.0	10.4	16.3	0.00006
KB 3.1	human endocervical adenocarcinoma (AC)	28.3	17.2	85.4	44.0	30.1	0.00079
PC-3	human prostate AC	23.7	9.9	n.d.	44.0	25.6	0.00008
SK-OV-3	human ovary AC	56.7	7.3	n.d.	66.0	33.1	0.00034
MCF-7	human breast AC	9.7	5.1	n.d.	8.8	7.8	0.00012
A431	human squamous AC	8.7	1.1	n.d.	5.7	16.3	0.00005
A549	human lung carcinoma	20.0	1.4	n.d.	17.0	27.1	0.00008

^1^ epothilone B (1mg/mL).

## Supplementary Materials

The following are available online at https://www.mdpi.com/2218-273X/10/5/805/s1: Protocol: Antimicrobial Activity Assay. Protocol: Cytotoxicity Assay. Figure S1. HPLC-UV/Vis chromatograms at 210 nm and DAD and HR-ESI-MS(+) traces of viridistratins A−C (**1**−**3**) and truncatones A+C (**4**, **5**). Figure S2. ECD spectrum of truncatone A (**4**). Figure S3. HPLC-UV/Vis chromatogram at 210 nm of the crude extract of *A. viridistratum*. Figures S4−S8. 1D and 2D NMR spectra of viridistratin A (**1**). Figures S9−S14. 1D and 2D NMR spectra of viridistratin B (**2**). Figures S15−S19. 1D and 2D NMR spectra of viridistratin C (**3**).
