# Supplementary material for: Viridistratins A−C, Antimicrobial and Cytotoxic Benzo[j]fluoranthenes from Stromata of Annulohypoxylon viridistratum (Hypoxylaceae, Ascomycota)
Source: Biomolecules. 2020 May 23;10(5):805. doi: 10.3390/biom10050805 (PMC7277860; doi:10.3390/biom10050805)
Supplement: Supplementary file 1 [file biomolecules-10-00805-s001.pdf]

Supporting Information

Viridistratins A−C, Antimicrobial and Cytotoxic Benzo[*j*]fluoranthenes from Stromata of *Annulohypoxylon viridistratum* (Hypoxylaceae, Ascomycota)

Kevin Becker [1,2], Anna-Charleen Wessel [1], J. Jennifer Luangsa-ard [3], Marc Stadler [1,2,*]

[1] Department of Microbial Drugs, Helmholtz Centre for Infection Research GmbH (HZI), Inhoffenstraße 7, 38124 Braunschweig, Germany; kevin.becker@helmholtz-hzi.de (K.B.), anna-charleen.wessel@helmholtz-hzi.de (A.C.W.)

[2] German Centre for Infection Research Association (DZIF), Partner site Hannover-Braunschweig, Inhoffenstraße 7, 38124 Braunschweig, Germany

[3] National Center for Genetic Engineering and Biotechnology (BIOTEC), 113 Thailand Science Park, Phahonyothin Rd., Khlong Nueng, Khlong Luang, Pathum Thani 12120, Thailand; jajen@biotec.or.th (J.J.L.)

* Correspondence: marc.stadler@helmholtz-hzi.de; Tel.: Tel.: +49-531-6181-4240; Fax: +49-531-6181-9499

Protocol: Antimicrobial Activity Assay

The assay was conducted as a minimum inhibitory concentration (MIC) assay in 96-well round-bottom microtiter plates using the parameters summarized in Table S1 and as already described in [S1].

Stocks of the test organisms were generated by growing the organisms overnight in 50 mL shaking flasks filled with 25 mL of the growth medium at 140 rpm (for media and temperatures see Table S1). If the organisms were well grown the next day, which was checked by occurrence of an optical density (OD)>30 of the suspension ($OD_{600\ nm}$ for bacteria, $OD_{548\ nm}$ for fungi and *M. smegmatis*), aliquots of these were stored in 1.5 mL reaction tubes in a freezer at −80 °C for up to 12 months. Upon use, aliquots were unthawed and the OD of the suspension measured and adjusted by diluting with the respective growth medium. $OD_{600\ nm}$ was adjusted to 0.01 and $OD_{548\ nm}$ to 0.1.

Subsequently, 150 µL of the adjusted suspensions were added to all wells of a 96-well microtiter plate (one test organism per plate). In row A, additional 130 µL of suspensions plus 20 µL of the test compounds (1 mg/mL) and the controls (one compound/column) were added. MeOH as well as MeOH:DMSO 9+1 were used as negative controls, while different positive controls (references) were used for the test organisms (see Table S1). Then, starting from row A, 150 µL of the suspension were transferred to the next row, the contents thoroughly mixed, and 150 µL transferred to the following row. The remaining 150 µL after row H were discarded. This resulted in a serial dilution of the test compounds, ranging from 66.7 µg/mL in row A to 0.52 µg/mL in row H.

The microtiter plates were then incubated overnight on a microplate shaker at 800 rpm at 30 or 37 °C (see Table S1) and were visually evaluated the next day. The MIC is defined as the lowest concentration were no growth of the test organism was observed. A lower MIC thus corresponds to a higher antimicrobial activity of the test compound.

Table S1: MIC assay experiment parameters

test organism	strain No.	growth medium	incubation temp. [°C]	positive control (reference)
Bacillus subtilis	DSM10	MHB [1]	30	oxytetracyclin 1.0 mg/mL
Staphylococcus aureus	DSM346	MHB [1]	30	oxytetracyclin 0.1 mg/mL
Micrococcus luteus	DSM1790	MHB [1]	30	oxytetracyclin 0.1 mg/mL
Chromobacterium violaceum	DSM30191	MHB [1]	30	oxytetracyclin 0.1 mg/mL
Escherichia coli	DSM1116	MHB [1]	37	oxytetracyclin 0.1 mg/mL
Pseudomonas aeruginosa	PA14	MHB [1]	37	gentamicin 0.1 mg/mL
Mycolicibacterium smegmatis	ATCC700084	7H9+ADC [2]	37	kanamycin 0.1 mg/mL
Candida albicans	DSM1665	MYC [3]	30	nystatin 1.0 mg/mL
Schizosaccharomyces pombe	DSM70572	MYC [3]	30	nystatin 1.0 mg/mL
Mucor hiemalis	DSM2656	MYC [3]	30	nystatin 1.0 mg/mL
Pichia anomala	DSM6766	MYC [3]	30	nystatin 1.0 mg/mL
Rhodotorula glutinis	DSM10134	MYC [3]	30	nystatin 1.0 mg/mL

[1] MHB: Müller-Hinton Broth (SN X927.1, Carl Roth GmbH, Karlsruhe, Germany)

[2] 7H9+ADC: Middlebrook 7H9 Broth Base + Middlebrook ADC Growth Supplement (SN M0678+M0553, Merck, Darmstadt, Germany)

[3] MYC: 1 % w/v, bacto peptone, 1% w/v yeast extract, 2 % w/v glycerol, pH 6.3

[S1] Helaly, S.E.; Ashrafi, S.; Teponno, R.B.; Bernecker, S.; Dababat, A.A.; Maier, W.; Stadler, M. Nematicidal Cyclic Lipodepsipeptides and a Xanthocillin Derivative from a Phaeosphariaceous Fungus Parasitizing Eggs of the Plant Parasitic Nematode *Heterodera filipjevi*. *J. Nat. Prod*. **2018**, *81*, 2228–2234.

Protocol: Cytotoxicity Assay

The assay was conducted in 96-well flat-bottom microtiter plates using the parameters summarised in Table S2 and as described in [S2].

Cell lines L929 and KB 3.1 were incubated at 37 °C under 10 % CO_2 in Gibco™ DMEM medium (Thermo Fisher Scientific, Waltham, MA, USA) supplemented with 10 % FBS. A microtiter plate was filled with 120 µL of this suspension (50,000/mL) in each well.

Seperately, another microtiter plate was filled with 100 µL of growth medium in each well. Then, 50 µL of the test compound solutions (1 mg/mL) were given to wells of the first column in two replicates (one compound per row). Cells without additives, MeOH, and MeOH:DMSO 9+1 were used as negative controls. Starting from the first column, 50 µL of the solutions were gradually transferred to the next column, the contents thoroughly mixed, and 50 µL transferred to the following column. This created a serial dilution of the test compounds ranging from 333 µg/mL to 1.9×10^{-3} µg/mL. The remaining 50 µL after column twelve were discarded. From this microtiter plate, 60 µL of the solutions from 111 µg/mL to 1.9×10^{-3} µg/mL were given to the first plate containing 120 µL of the cell suspensions (*i.e.* the highest concentration 333 µg/mL was not used). This resulted in final compound concentrations ranging from 37 µg/mL to 0.6×10^{-3} µg/mL.

After 5 days of incubation under the aforementioned incubation conditions, the half maximum inhibitory concentrations (IC_{50}) were determined using a colorimetric tetrazolium dye MTT assay [S3]. For this, 20 µL of a 5 mg/mL solution of 3-(4,5-dimethyl-2-thiazolyl)-2,5-diphenyl-2*H*-tetrazolium bromide (MTT) were added to each well and incubated for two hours at 37 °C. Then, the microtiter plate was centrifuged (3,000 rpm, 5 min) and the supernatant removed by holding the plate upside-down and gentle shaking. Afterwards, the wells were washed using 100 µL of phosphate buffered saline (PBS). The plate was again centrifuged and the supernatant removed as described before. Then, 100 µL of an isopropanol:HCl solution (1L isopropanol+4 mL HCl 37 % w/v) were added to the wells. After incubating for 10 min at ambient temperature, the absorption of the wells at 595 nm was measured with an Infinite® 200 Pro microplate reader (TECAN, Männedorf, Schweiz).

The absorption values of the cells without additives were averaged and set to 100 % cell viability. Then, the means of absorption of the two compound replicates were set in relation to the blank media. These percentage values were plotted against the concentration range (37 µg/mL to 0.6×10^{-3} µg/mL). The IC_{50} value was read from the plot (in µg/mL) and the units conversed to µM.

If effects were observed with cell lines L929 and KB 3.1 (IC_{50} < 50 µM), the other cell lines were tested using the same protocol.

Table S2: Cytotoxicity assay experiment parameters

cell line	type	No.	growth medium
L929	mouse fibroblasts	ACC 2	DMEM [1]
			+ 10 % FBS [2]
KB 3.1	human endocervical adenocarcinoma (AC)	ACC 158	DMEM [1]
			+ 10 % FBS [2]
PC-3	human prostate AC	ACC 465	F-12K Nutmix [3]
			+ 10 % FBS [2]
SK-OV-3	human ovary AC	n/a	McCoys 5a [4]
			+ 10 % FBS [2]
MCF-7	human breast AC	ACC 115	RPMI 1640 [5]
			+ 10 % FBS [2]
			+ 1 % MEMNEAA [6]
			+ 1.25 mL/500 mL insulin [7]
A431	human squamous AC	ACC 91	RPMI 1640 [4]
			+ 10 % FBS [2]
A549	human lung carcinoma	ACC 107	DMEM [1]

[1] DMEM: Dulbecco's Modified Eagle Medium (SN 61965026, Thermo Fisher Scientific, Waltham, MA, USA)

[2] FBS: Fetal Bovine Serum (SN 10500064, Thermo Fisher Scientific)

[3] F-12K Nutmix: Ham's F-12K (Kaign's) Medium (SN 21127022, Thermo Fisher Scientific)

[4] McCoys 5a: McCoy's 5a (modified) Medium (SN 26600023, Thermo Fisher Scientific)

[5] RPMI 1640: RPMI 1640 Medium (SN 21875091, Thermo Fisher Scientific)

[6] MEMNEAA: MEM Non-Essential Amino Acids Solution 100× (SN 11140035, Thermo Fisher Scientific)

[7] Insulin: Human Recombinant Insulin, Zinc Solution (SN 12585014, Thermo Fisher Scientific)

[S2] Sandargo, B.; Michehl, M.; Praditya, D.; Steinmann, E.; Stadler, M.; Surup, F. Antiviral Meroterpenoid Rhodatin and Sesquiterpenoids Rhodocoranes A-E from the Wrinkled Peach Mushroom, *Rhodotus palmatus. Org. Lett.* **2019,** *21*, 3286–3289.

[S3] Mosmann, T. Rapid colorimetric assay for cellular growth and survival: Application to proliferation and cytotoxicity assays. *J. Immunol. Methods* **1983**, *65*, 55–63.

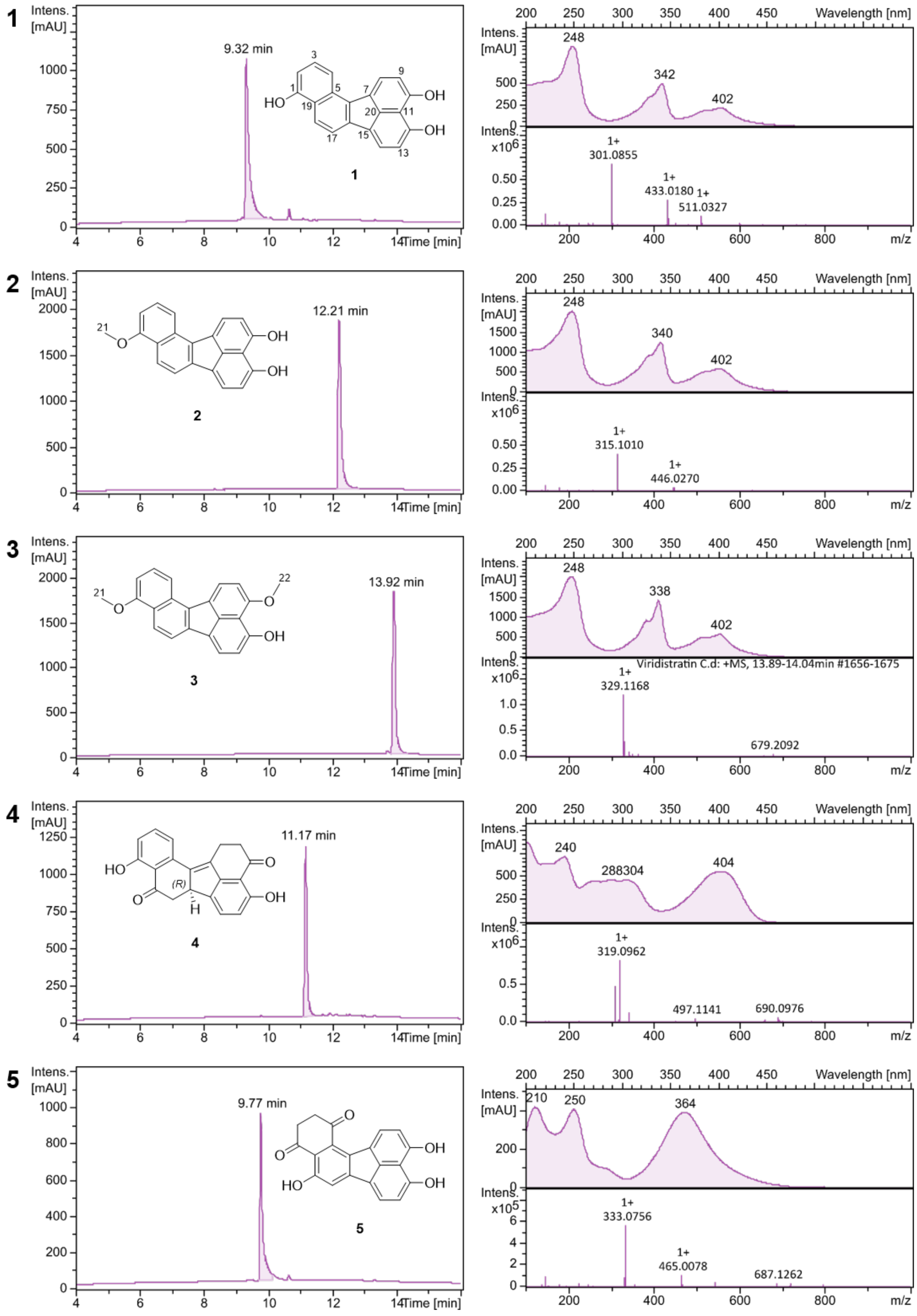

Figure S1: HPLC-UV/Vis chromatograms at 210 nm, DAD and HR-ESI-MS(+) traces of viridistratins A−C (**1**−**3**) and truncatones A+C (**4**, **5**).

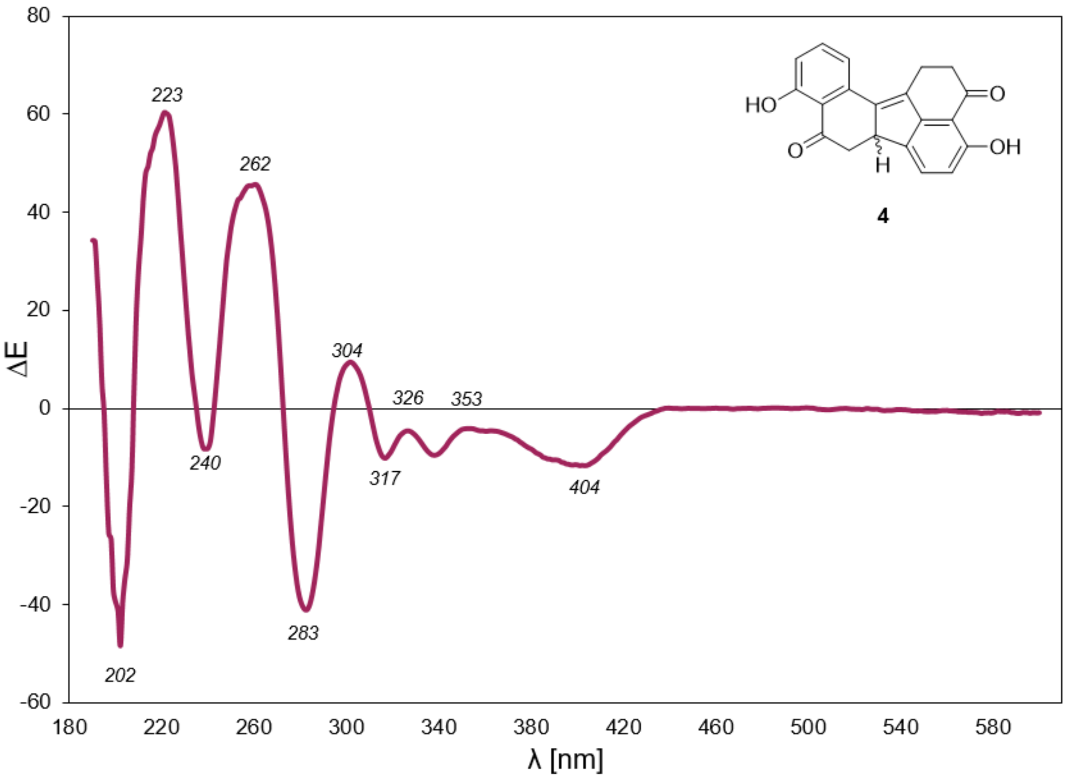

Figure S2: ECD spectrum of truncatone A (**4**). Italic numbers: important maxima/minima of the spectrum.

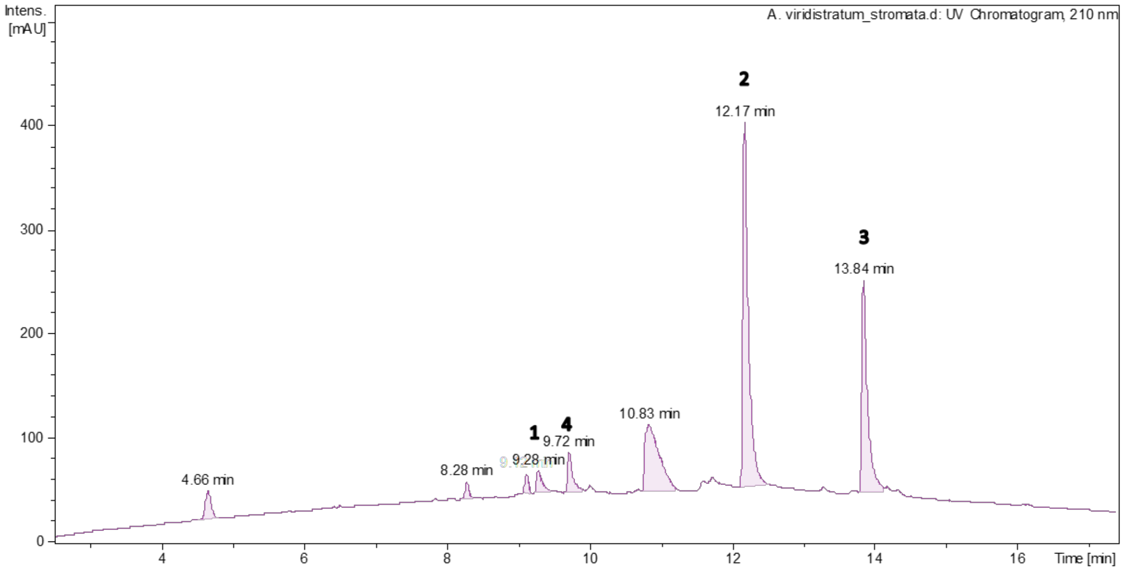

Figure S3: HPLC-UV/Vis chromatogram at 210 nm of the crude extract of *A. viridistratum*. **1**–**3**: viridistratins A–C, **4**: truncatone C. Truncatone A (**5**) was not found in the crude extracts.

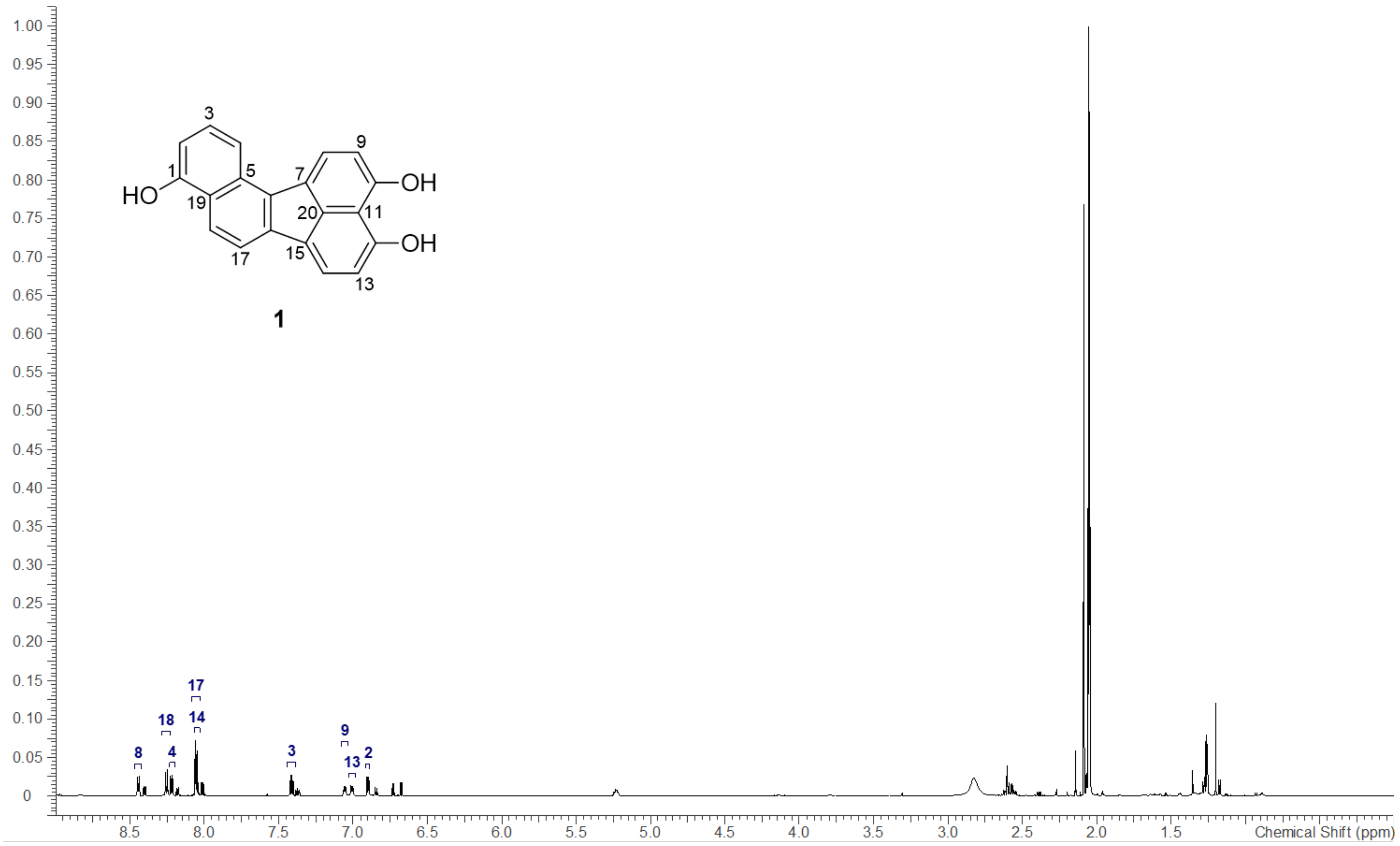

Fig. S4: ^{1}H NMR spectrum (500 MHz, acetone-*d*6) of viridistratin A (**1**).

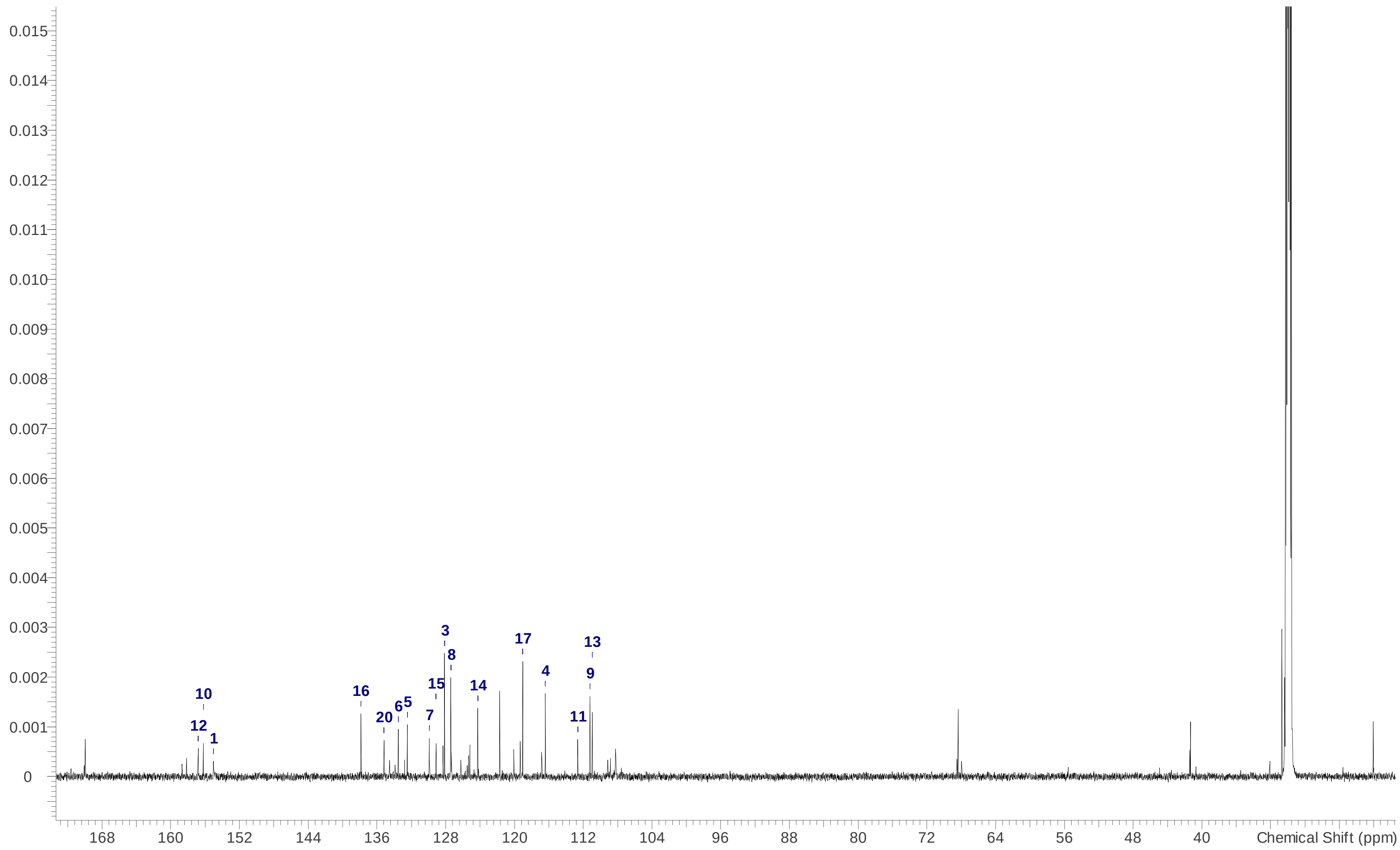

Fig. S5: ^{13}C NMR spectrum (125 MHz, acetone-*d*$_6$) of viridistratin A (**1**).

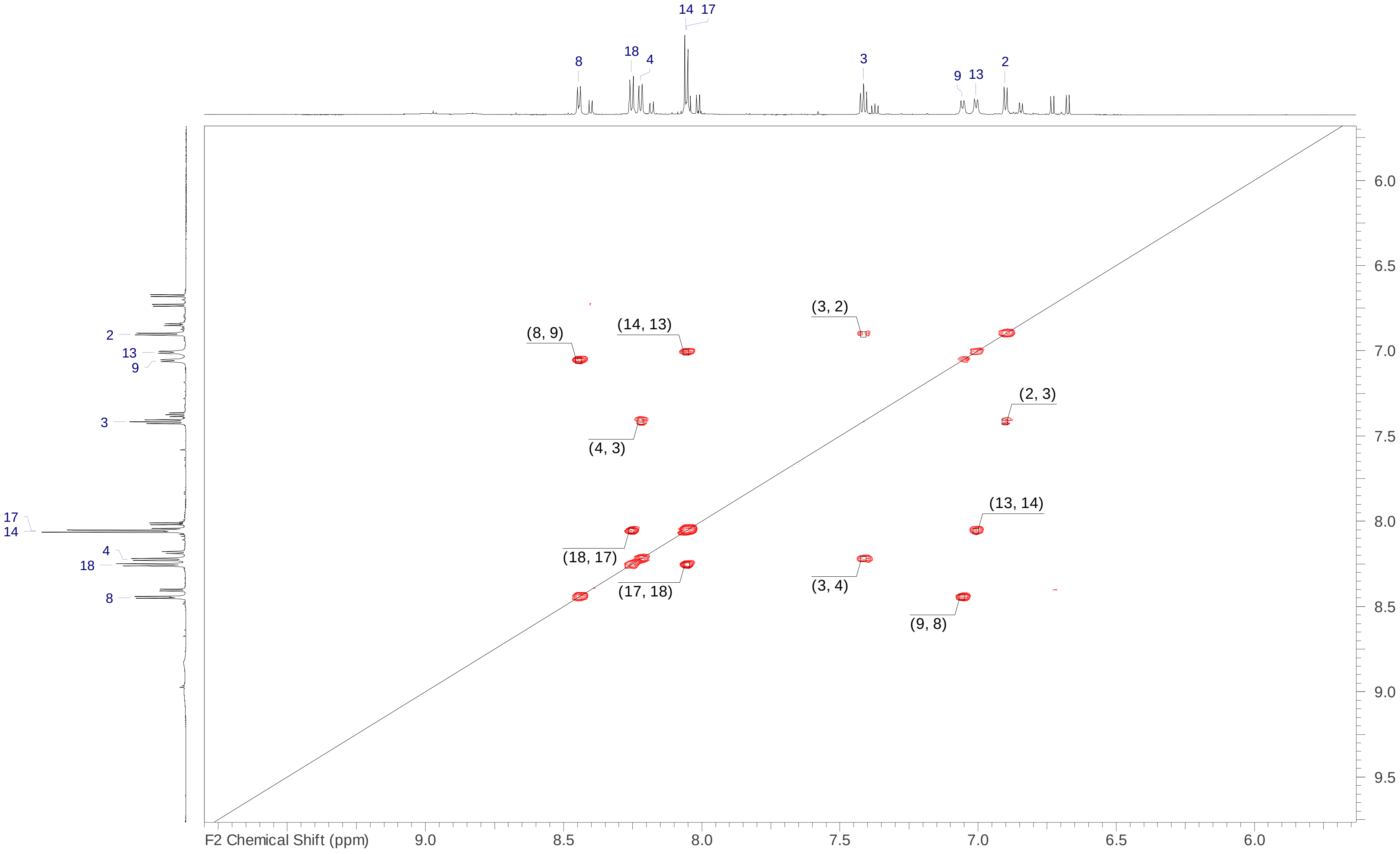

Fig. S6: ^{1}H/^{1}H COSY spectrum (500 MHz, acetone-d_6) of viridistratin A (**1**).

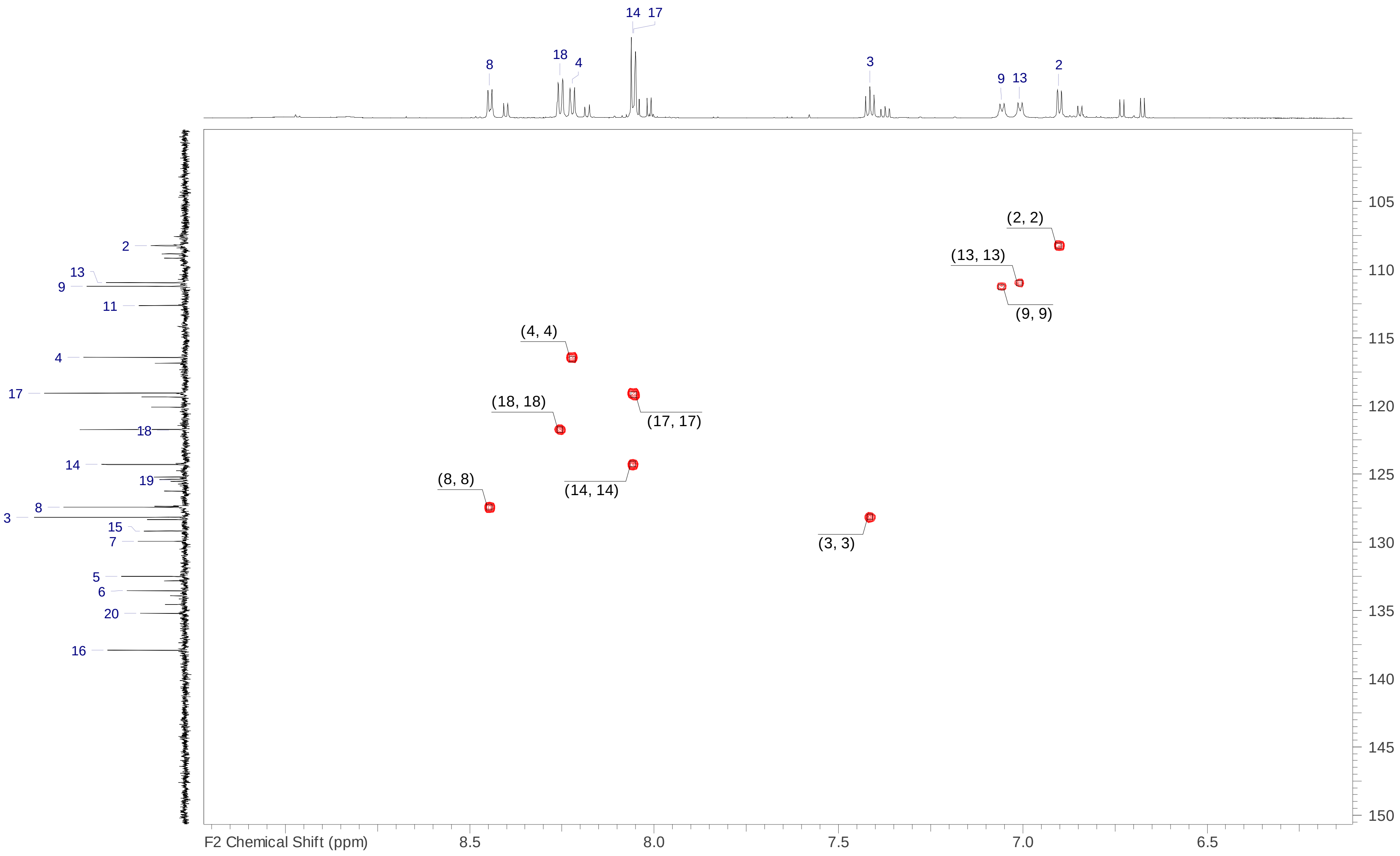

Fig. S7: ^{1}H/^{13}C HSQC spectrum (500 MHz, acetone-d_6) of viridistratin A (**1**).

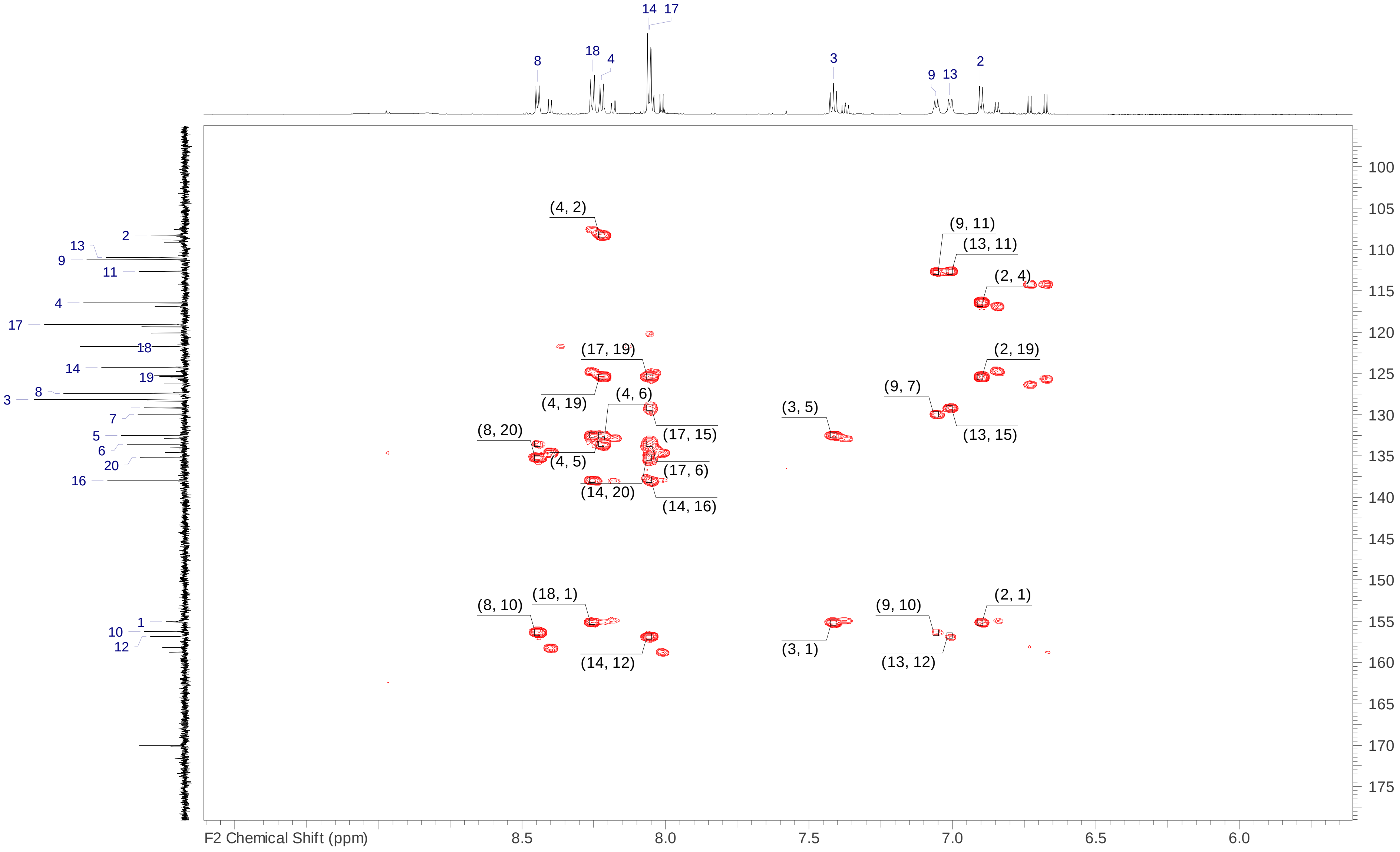

Fig. S8: $^{1}H/^{13}C$ HMBC spectrum (500 MHz, acetone-d_6) of viridistratin A (**1**).

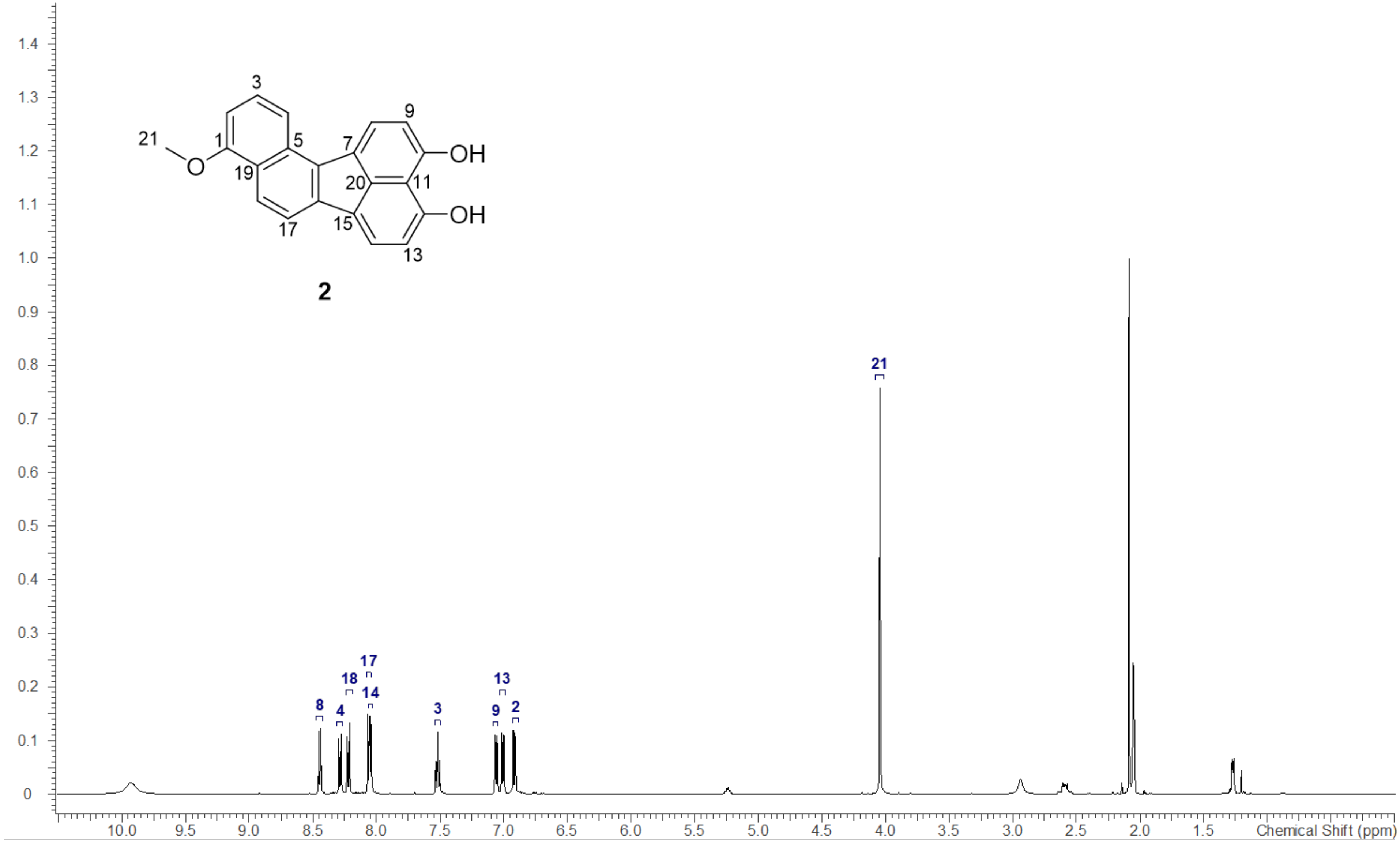

Fig. S9: ^{1}H NMR spectrum (500 MHz, acetone-d_6) of viridistratin B (**2**).

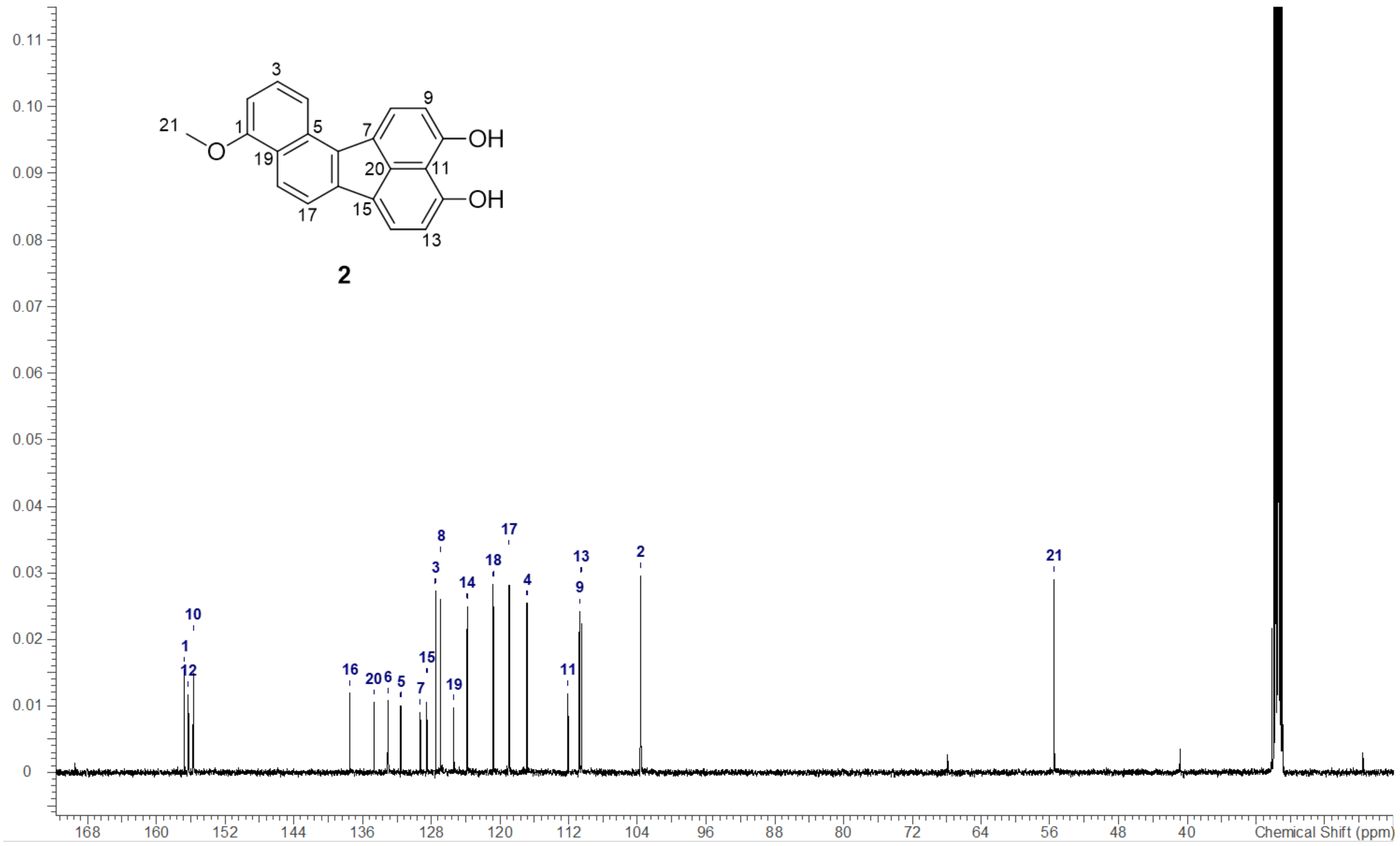

Fig. S10: ^{13}C NMR spectrum (500 MHz, acetone-d_6) of viridistratin B (**2**).

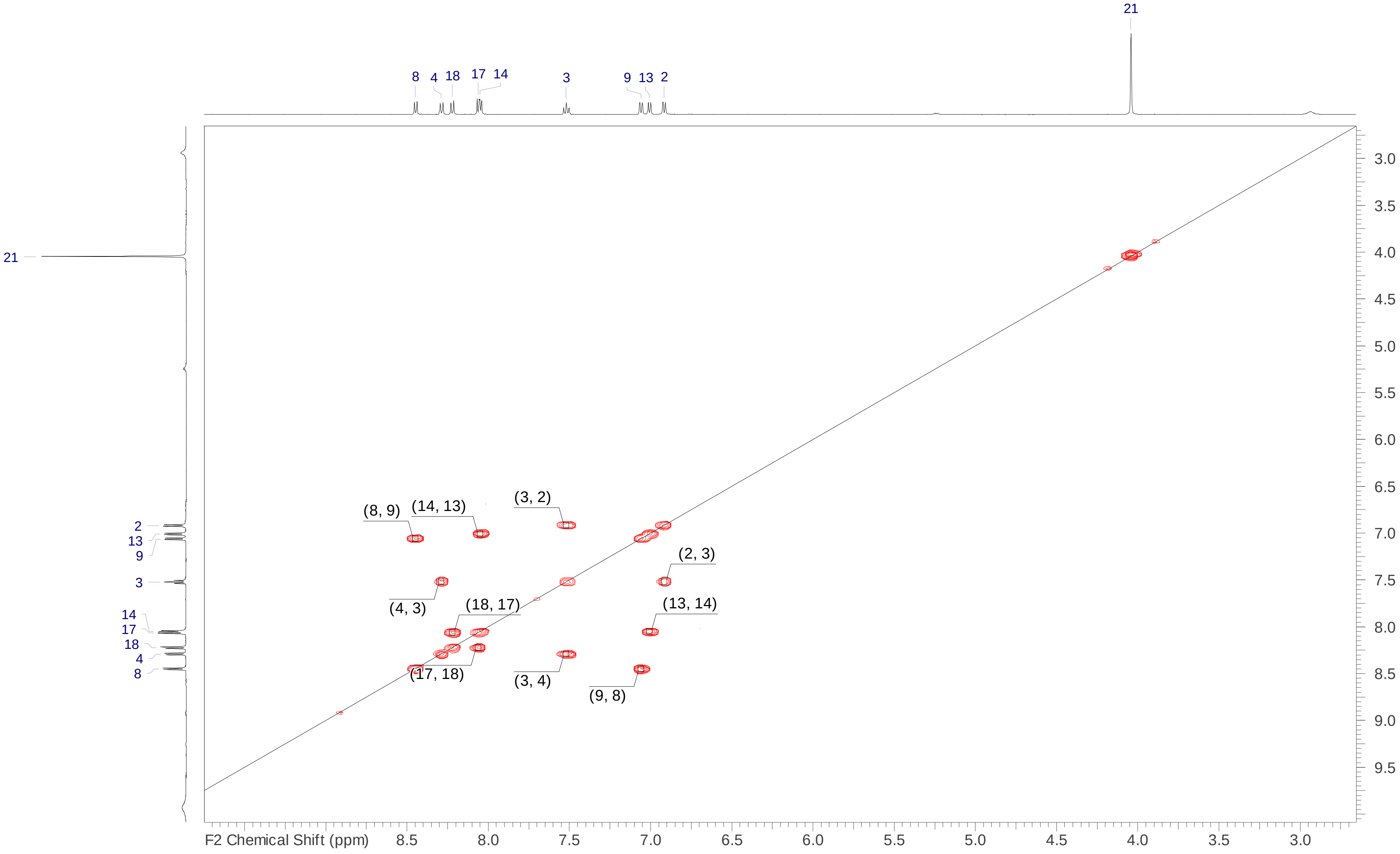

Fig. S11: ^{1}H/^{1}H COSY spectrum (500 MHz, acetone-d_6) of viridistratin B (**2**).

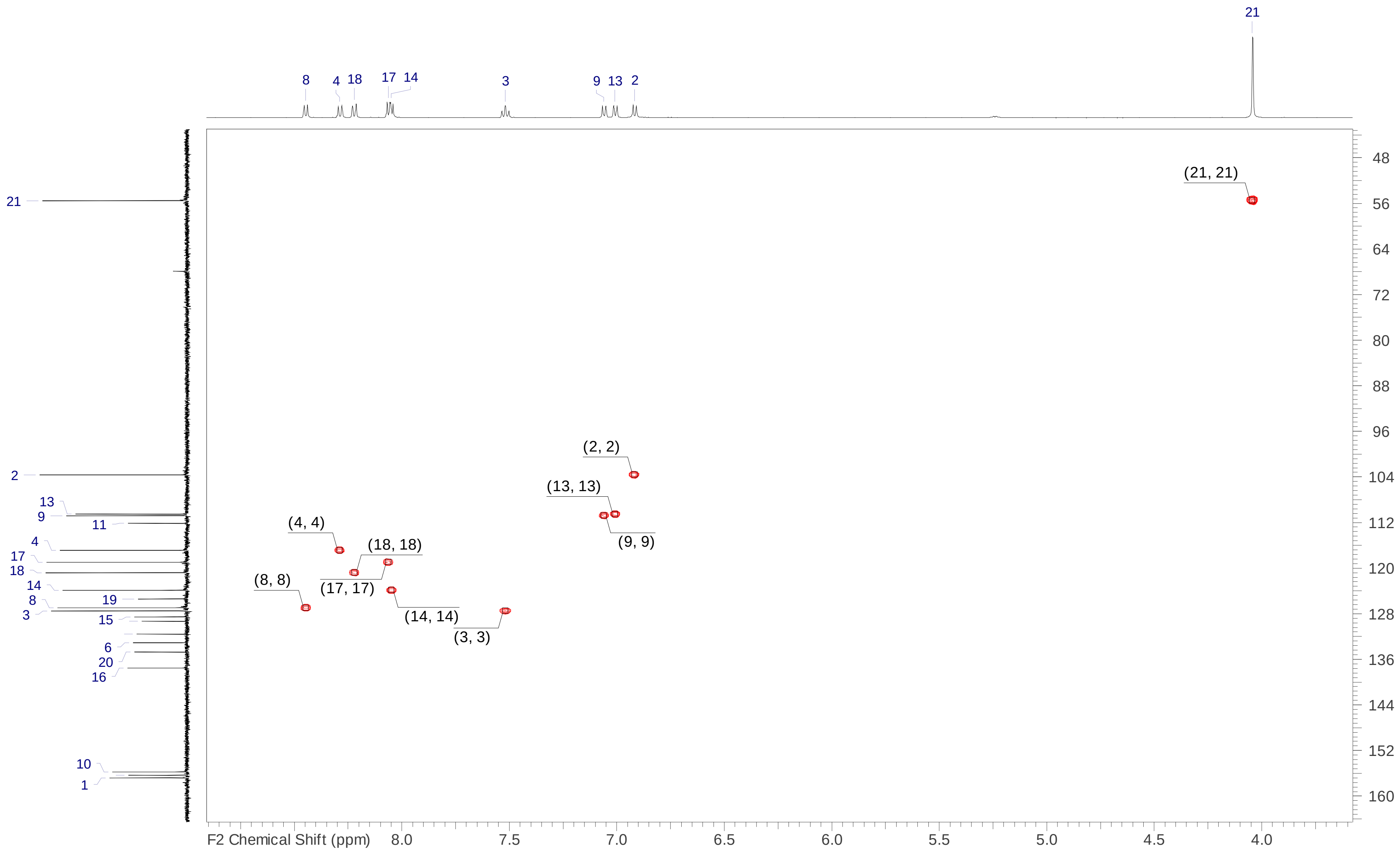

Fig. S12: $^{1}H/^{13}C$ HSQC spectrum (500 MHz, acetone-d_6) of viridistratin B (**2**).

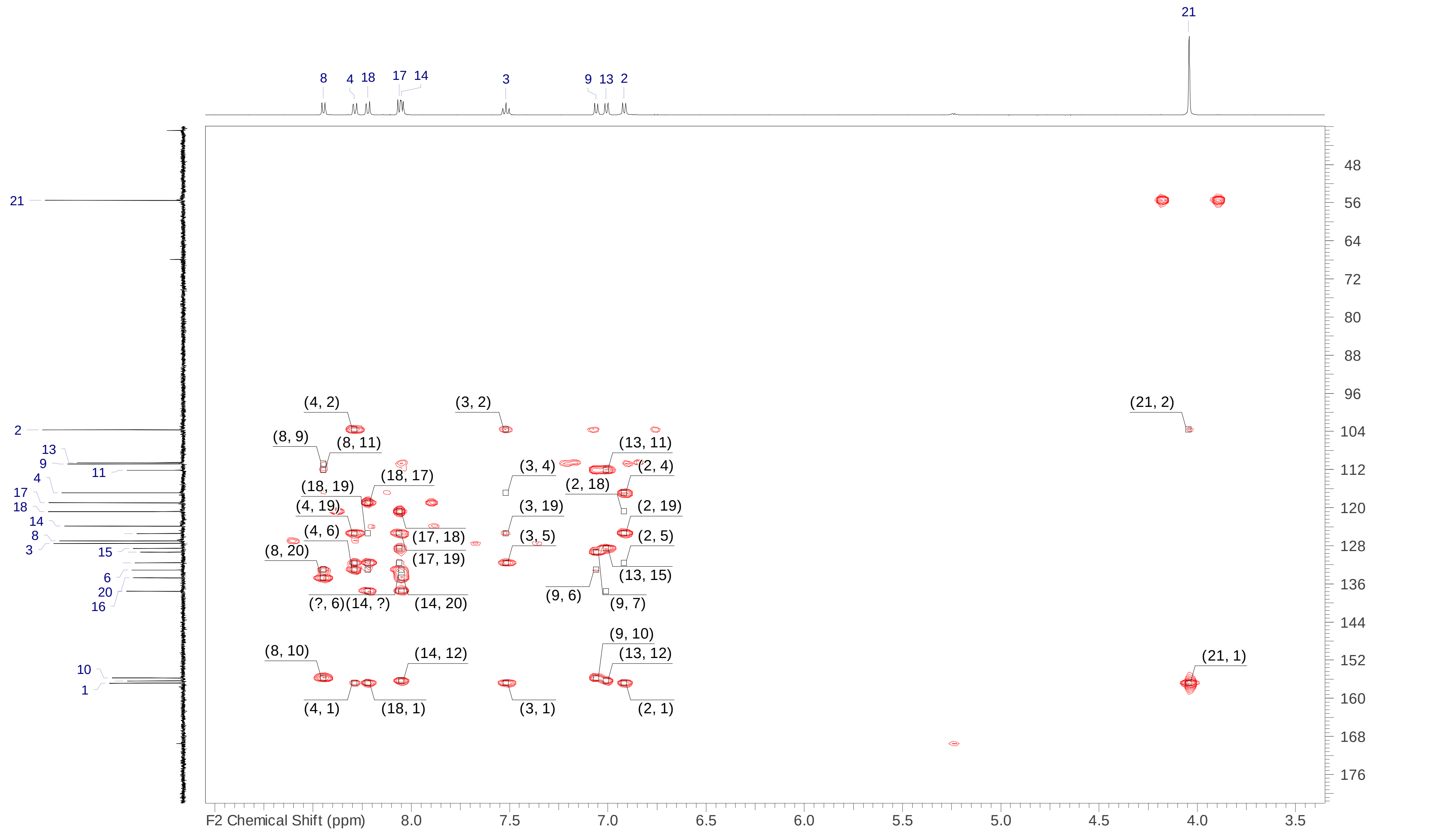

Fig. S13: $^{1}H/^{13}C$ HMBC spectrum (500 MHz, acetone-d_6) of viridistratin B (**2**).

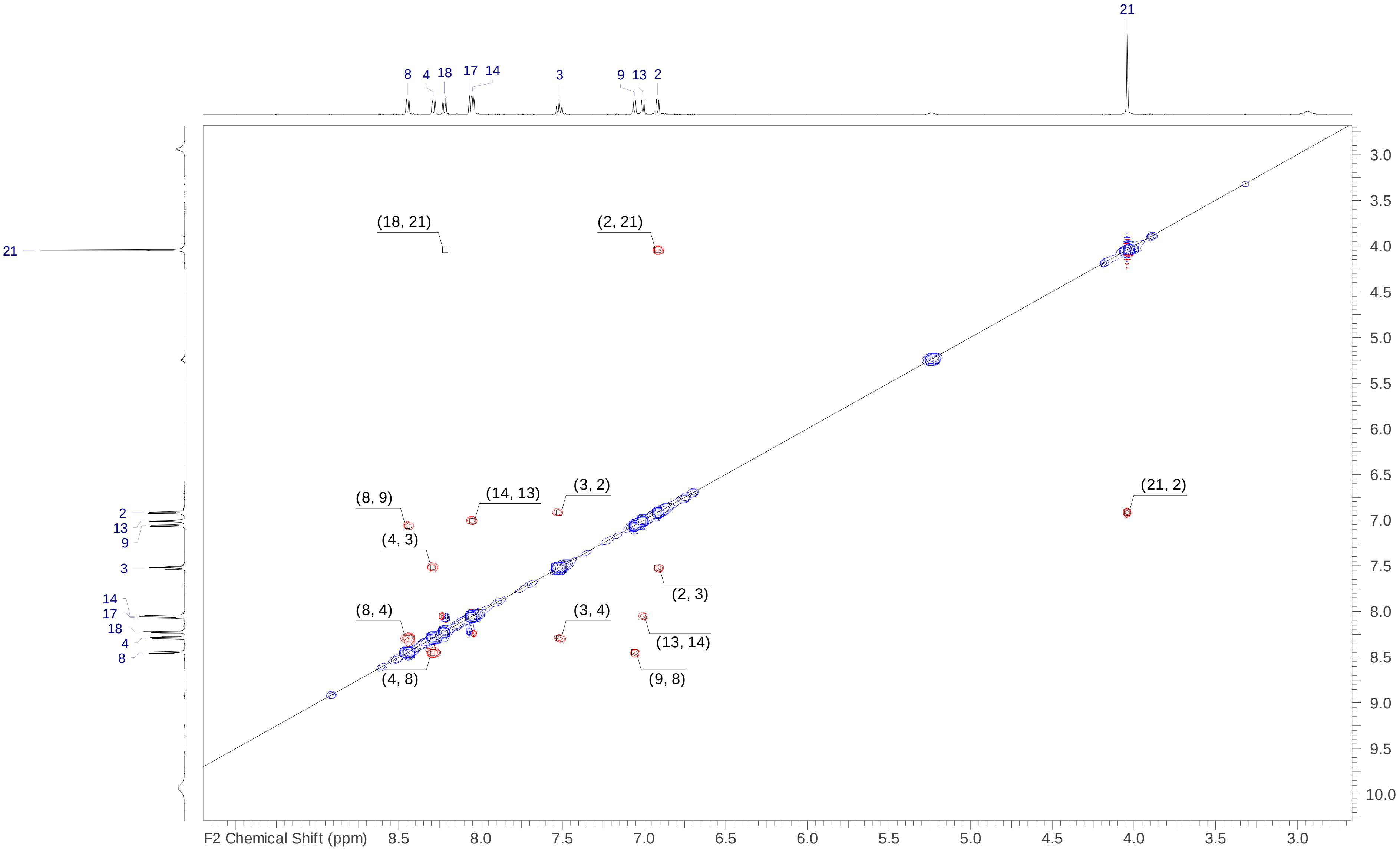

Fig. S14: ROESY spectrum (500 MHz, acetone-d_6) of viridistratin B (**2**).

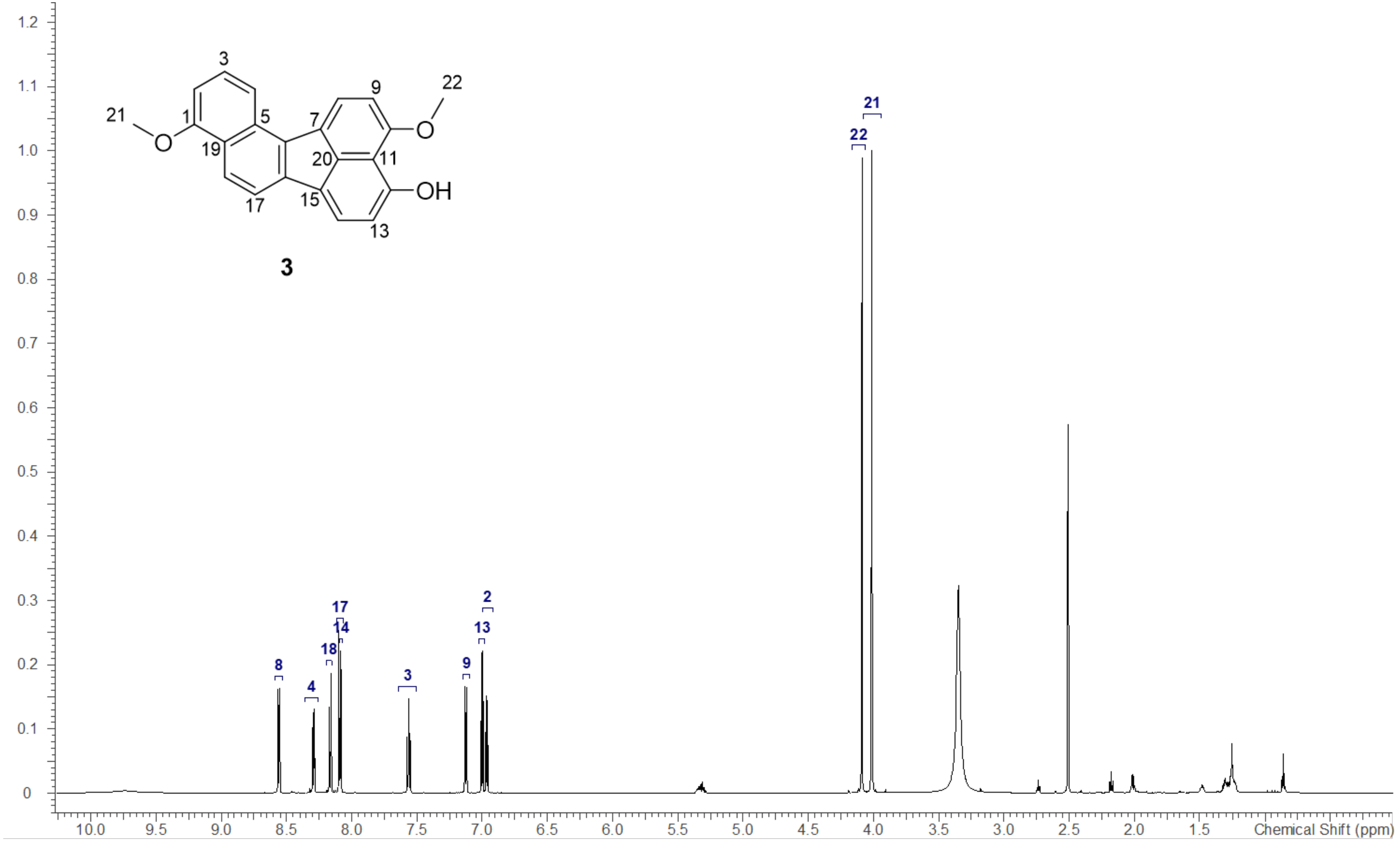

Fig. S15: ^{1}H NMR spectrum (500 MHz, DMSO-*d*₆) of viridistratin C (**3**).

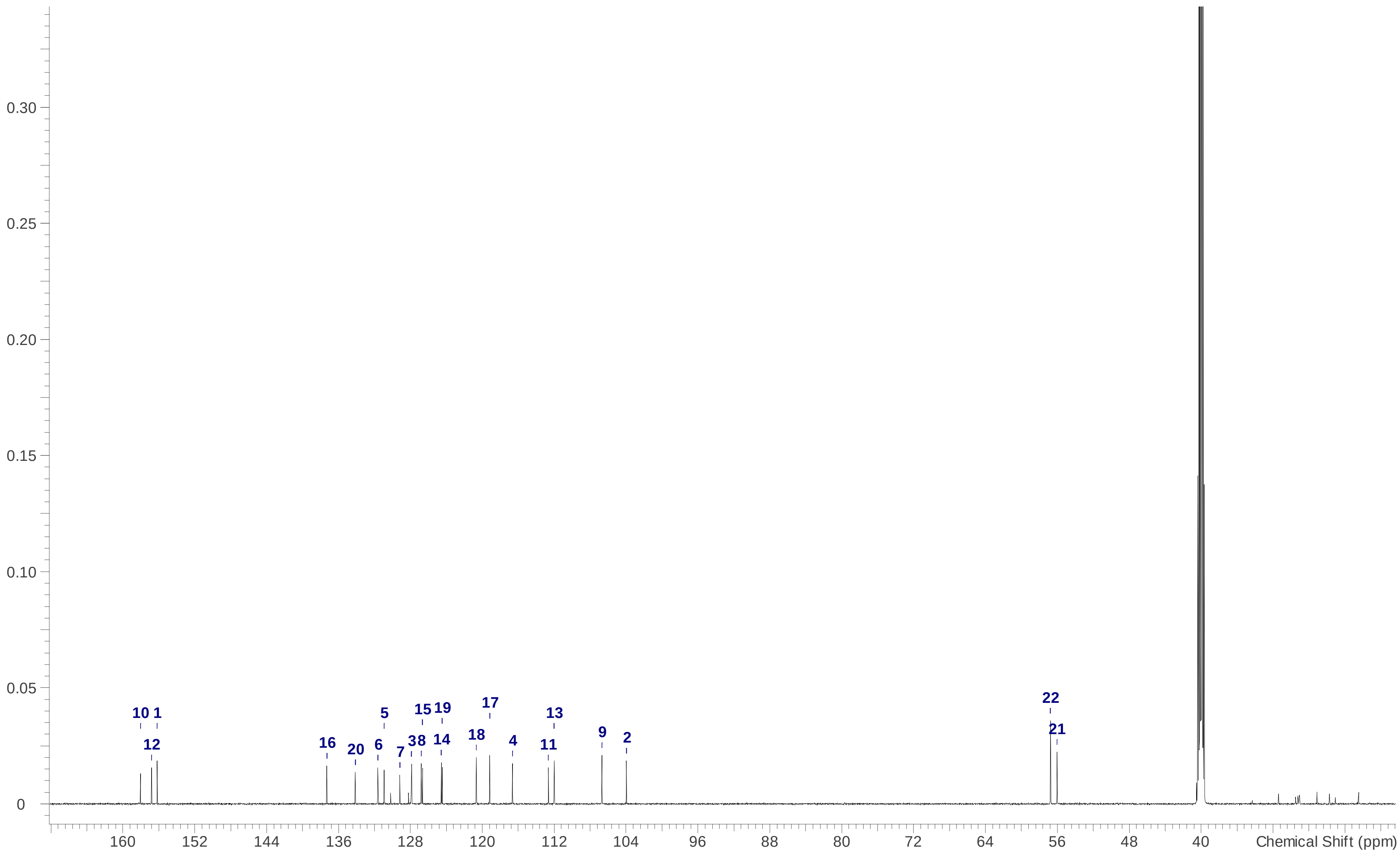

Fig. S16: ^{13}C NMR spectrum (125 MHz, DMSO-d_6) of viridistratin C (**3**).

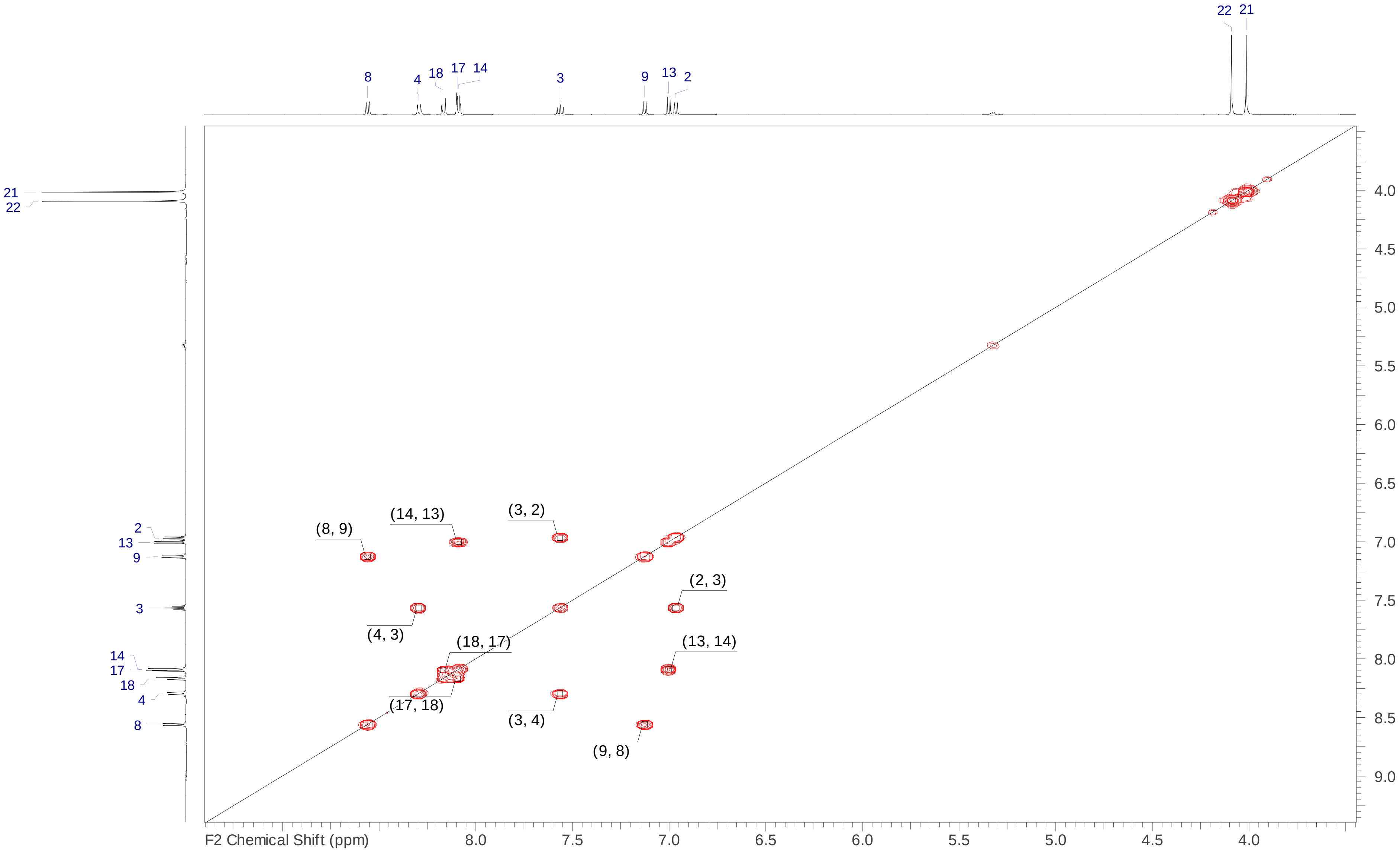

Fig. S17: $^1H/^1H$ COSY spectrum (500 MHz, DMSO-*d*$_6$) of viridistratin C (**3**).

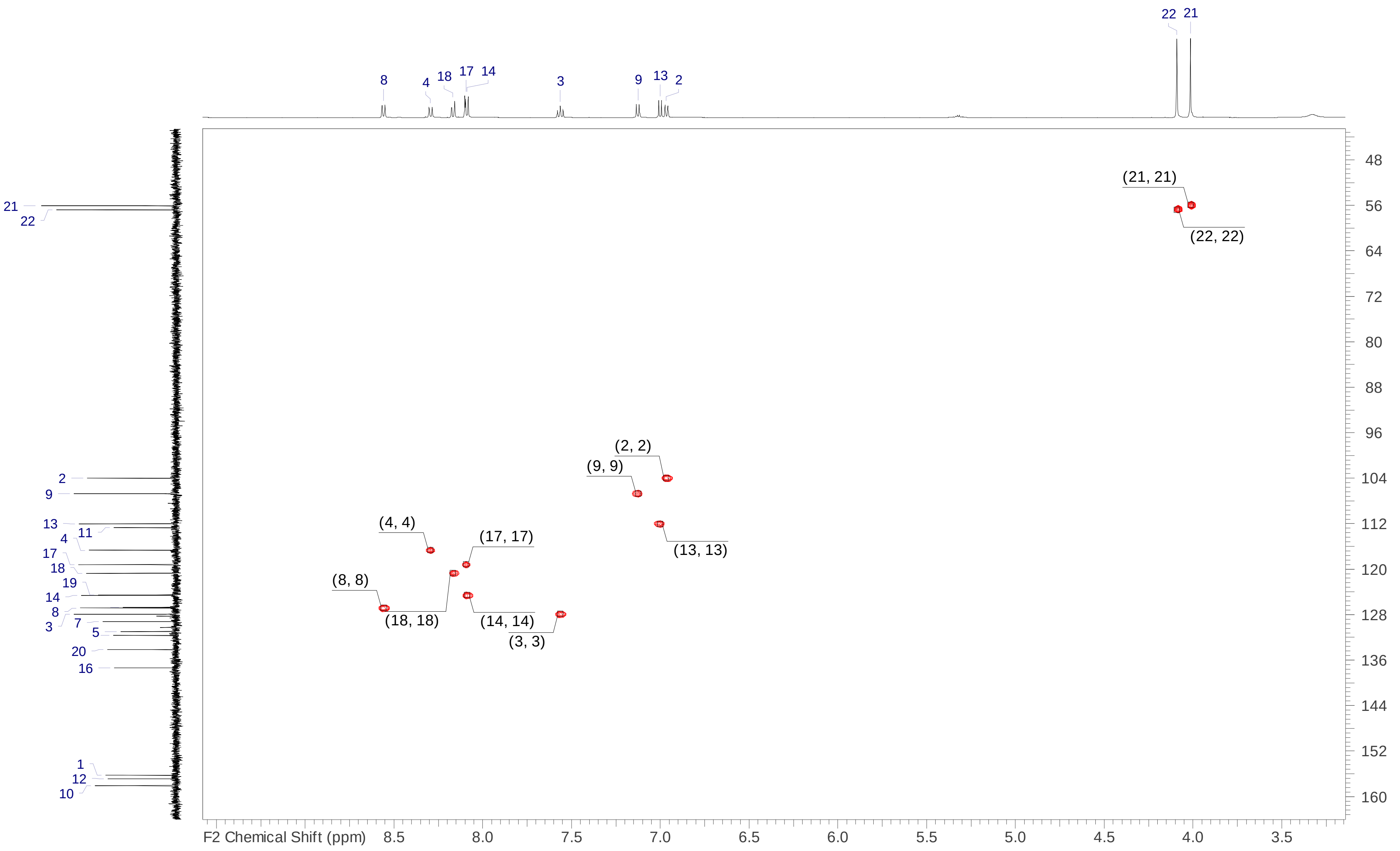

Fig. S18: $^{1}H/^{13}C$ HSQC spectrum (500 MHz, DMSO-d_6) of viridistratin C (**3**).

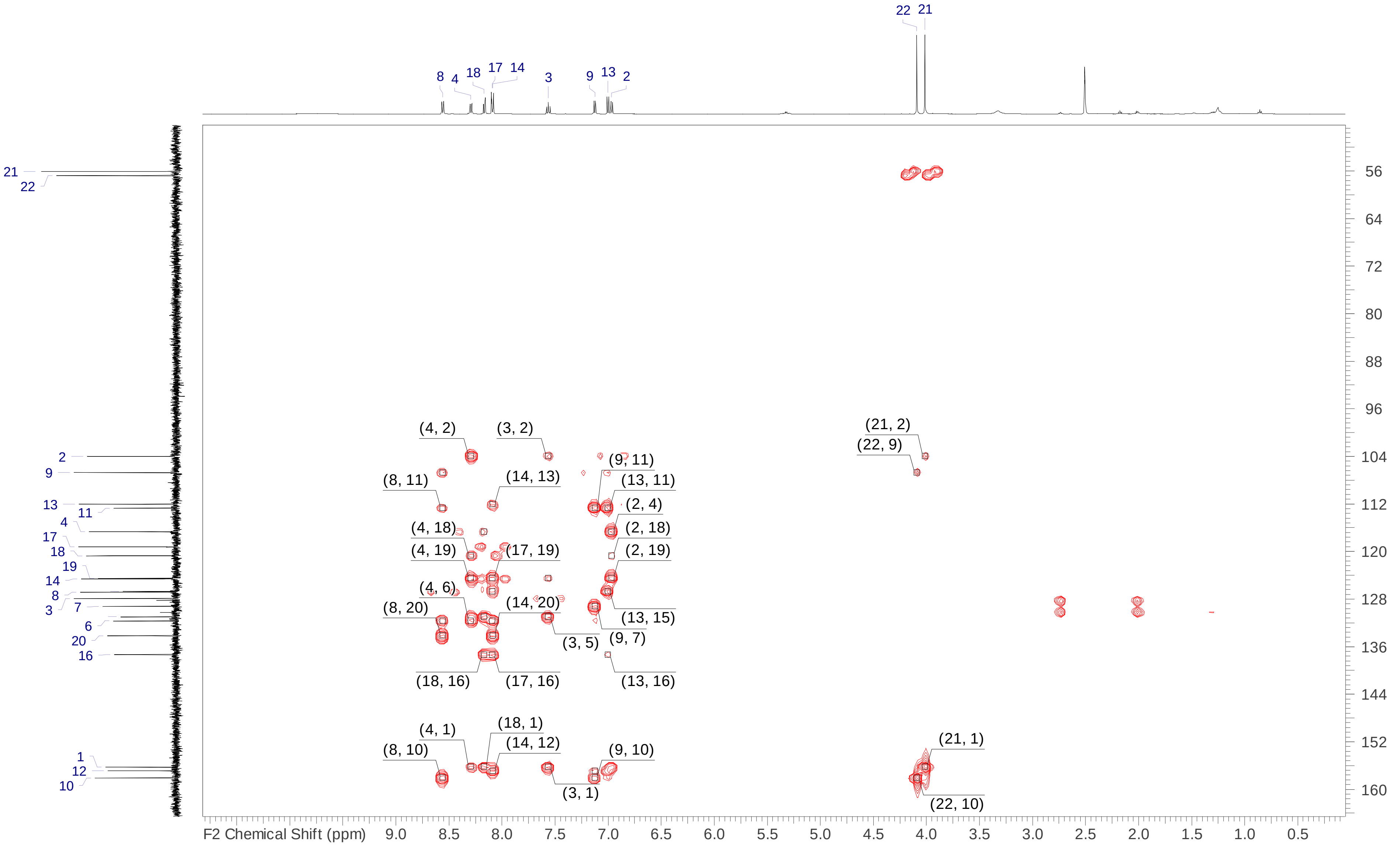

Fig. S19: $^{1}H/^{13}C$ HMBC spectrum (500 MHz, DMSO-d_6) of viridistratin C (**3**).
